# Fire as a regeneration filter in the Arctic: effects of heat and smoke on seed germination in 25 common plant species

**DOI:** 10.1093/aob/mcag236

**Published:** 2026-08-14

**Authors:** Jerónimo Vázquez-Ramírez, Margherita Tognela, Natasha de Vere, Barbara Gawlak, Julia Kemppinen, Daniel Kępski, David Kniha, Simone Iris Lang, Petr Macek, Maija Sujala, Otso Suominen, Anne Tolvanen, Brandon Samuel Whitley, Sergey Rosbakh

**Affiliations:** Department of Biology, University of Copenhagen, Copenhagen 2100, Denmark; Department of Plant and Environmental Sciences, University of Copenhagen, Copenhagen 1871, Denmark; Department of Earth and Environmental Sciences, University of Pavia, Pavia 27100, Italy; Natural History Museum of Denmark, University of Copenhagen, Copenhagen 1353, Denmark; Institute of Geophysics, Polish Academy of Sciences, Warsaw 01-452, Poland; Botany and Mycology Unit, University of Helsinki, Helsinki 00170, Finland; Department of Organismal and Evolutionary Biology, Faculty of Biological and Environmental Sciences, University of Helsinki, Helsinki 00014, Finland; Institute of Geophysics, Polish Academy of Sciences, Warsaw 01-452, Poland; Division of Environment and Natural Resources, Norwegian Institute of Bioeconomy Research, Ås 1433, Norway; Department of Arctic Biology, The University Centre in Svalbard, Longyearbyen 9171, Norway; Institute of Hydrobiology, Biology Centre of Czech Academy of Sciences, České Budějovice 370 05, Czech Republic; Chair of Biodiversity and Nature Tourism, Estonian University of Life Sciences, Tartu 51006, Estonia; Kilpisjärvi Biological Station, University of Helsinki, Kilpisjärvi 99490, Finland; Kevo Research Station, Biodiversity Unit, University of Turku, Utsjoki 99980, Finland; Natural Resources Institute Finland, Helsinki 00791, Finland; Natural History Museum of Denmark, University of Copenhagen, Copenhagen 1353, Denmark; Department of Plant and Environmental Sciences, University of Copenhagen, Copenhagen 1871, Denmark; Institute of Earth System Sciences, Leibnitz University Hannover, Hannover 30419, Germany

**Keywords:** Arctic tundra, climate change, fire ecology, seed germination, smoke cues, heat shock, post-fire recruitment

## Abstract

**Background and Aims:**

The rapid warming of the Arctic is increasing the frequency, intensity and spatial extent of fires. Because fire has historically been rare in this region, most Arctic plant species are unlikely to have evolved traits that confer tolerance to fire, and the consequences for early life-history stages, such as seed germination, remain largely unknown. Here, we tested experimentally the effects of two key fire-related cues, heat shock and smoke exposure, on the germination traits of 25 widespread Arctic plant species.

**Methods:**

Seeds collected across the Arctic were subjected to four treatments: (1) high heat (110 °C for 4 min, simulating surface fire exposure); (2) low heat (50 °C for 10 min, simulating soil seed bank conditions); (3) smoke water produced from Arctic plant biomass; and (4) a combination of low heat and smoke water. We analysed germination responses at three levels: overall, across functional groups (forbs, graminoids and woody species) and at the species level.

**Key Results:**

The high-heat treatment almost completely inhibited germination, reducing the final percentage of germination from ∼78 to ∼2 %, with only three species showing any germination, two of them graminoids. Low-heat treatment produced no overall or functional-group changes in final germination but reduced germination speed and germination synchrony in 20 % of the species. Smoke water did not alter the final germination percentage but accelerated overall germination speed by ∼9–11 %.

**Conclusions:**

Together, these results suggest that most Arctic species are not adapted to survive the temperatures experienced at or near the soil surface during fires; however, buried seeds might remain viable after low heating and be stimulated by smoke, creating an ecological filter that favours species with persistent soil seed banks. As Arctic fires become more frequent and intense, seed-based regeneration will be likely to play a growing role in shaping post-fire recovery and will influence the diversity and composition of plant communities.

## INTRODUCTION

Wildfires are emerging as a rapidly intensifying disturbance across the Arctic (Descals *et al.*, 2022; Gosden *et al.*, 2022). Historically rare and typically of small spatial extent (Masrur *et al.*, 2017), Arctic fires are now increasing in frequency, intensity, severity and spatial coverage (French *et al.*, 2015; McCarty *et al.*, 2021). This phenomenon is driven mainly by the region warming nearly four times faster than the global average (Rantanen *et al.*, 2022) and by increasingly longer, drier and more lightning-prone growing seasons (He *et al.*, 2022). When they occur, Arctic fires burn vegetation and organic soils, disrupting carbon dynamics (Mack *et al.*, 2011), accelerating permafrost thaw (Virkkala *et al.*, 2025) and altering plant community structure, diversity and composition (Heim *et al.*, 2025).

In contrast to other high-latitude ecosystems, such as boreal forests, where fire is a natural and recurring ecological disturbance that shapes vegetation dynamics (Kasischke and Turetsky, 2006), fire has probably not been a recent ecological and evolutionary driver for most Arctic species owing to its rarity in the region. Consequently, emerging fire regimes can produce disruptive and divergent ecological outcomes, with multiple post-fire vegetation trajectories and alternative states (e.g. Chen *et al.*, 2021; Gaglioti *et al.*, 2021; Heim *et al.*, 2025). Although frequent Arctic fires can lead to graminoid-dominated communities (e.g. Bret-Harte *et al.*, 2013; Hollingsworth *et al.*, 2021), they can also lead to shifts towards shrub-dominated landscapes (Landhausser and Wein, 1993; Racine *et al.*, 2004; Jones *et al.*, 2013; Chen *et al.*, 2021; Liu *et al.*, 2022). These contrasting trajectories highlight the complexity of the recovery of Arctic vegetation after a fire, which appears to be shaped by the fire regime (i.e. frequency, intensity and severity; Heim *et al.*, 2025), vegetation type (e.g. low or high tundra; Gaglioti *et al.*, 2021) and site-specific abiotic factors (e.g. soil properties and nutrients, near-surface permafrost and soil moisture; Chen *et al.*, 2021).

Following a low-intensity fire event, Arctic vascular plant vegetation can recover to near pre-fire levels within 4–10 years (Heim *et al.*, 2025). Much of this initial recovery is attributed to plants that resprout from surviving below-ground structures (Racine *et al.*, 1987; Bret-Harte *et al.*, 2013), particularly after low-severity fires that leave surface moss and peat intact, favouring vegetative regeneration over seedling establishment (Frost *et al.*, 2020). However, deep-burning fires that consume the organic soil layer can create post-fire conditions where recruitment from seeds, either from the soil seed bank or via dispersal, is the primary source of vegetation recovery (Racine *et al.*, 2004, 1987; Bret-Harte *et al.*, 2013; Liu *et al.*, 2022).

Despite evidence that seed-based regeneration contributes to post-fire vegetation recovery, Arctic seed responses to fire remain poorly understood. More quantitative evidence is needed to determine the extent to which Arctic seeds tolerate fire-generated temperatures; whether exposure to smoke-derived compounds (e.g. butenolides; Light *et al.*, 2009) affects germination; or which species and functional groups might be fire sensitive versus fire tolerant. Studies from fire-prone ecosystems have shown that species belonging to genera that also occur in the Arctic, such as *Empetrum*, *Vaccinium*, *Luzula* or *Rumex*, can respond to heat and smoke cues (Granström and Schimmel, 1993; Bargmann *et al.*, 2014), suggesting that related Arctic species might also be responsive to fire-related cues. However, whether this is the case remains largely unknown and requires systematic empirical evaluation. This lack of data from Arctic ecosystems limits our ability to predict how emerging fire regimes will shape recruitment and vegetation dynamics in a warming Arctic.

Here, we address this knowledge gap by conducting a multi-species experimental test of Arctic seed responses to fire-related cues. We simulated key aspects of Arctic fire to examine how two heat-shock levels, representing soil surface (110 °C for 4 min) and subsurface (50 °C for 10 min) temperatures and smoke exposure, influence the germination traits of 25 widespread Arctic vascular plant species. These species represent a variety of functional groups (graminoids, forbs and woody plants), allowing us to detect ecologically meaningful variation in responses. Based on the historically low fire frequency in the Arctic, we hypothesised that: (H1) any heat shock will reduce overall germination; (H2) smoke exposure will not significantly affect germination; but (H3) species will differ in the magnitude and direction of their responses, potentially reflecting phylogenetic or functional trait patterns.

## MATERIALS AND METHODS

### Study species and seed collection

We selected 25 widespread Arctic plant species (Table 1) representing three functional groups: forbs (12), graminoids (4) and woody species (9, including trees, tall shrubs and dwarf shrubs). Seeds were collected during their natural dispersal period (July–September 2023 and 2024) from >20 individuals within a single population per species across multiple Arctic sites (Supplementary Data Table S1; Supplementary Data Fig. S1). After collection, seeds were air-dried at room temperature and transported in paper bags in ambient conditions to the laboratory in Copenhagen, Denmark. On arrival, seeds were cleaned and stored at 5 °C, 35 ± 4 % (mean ± s.d.) relative humidity and dark conditions until the germination experiment commenced in November 2024. Preliminary germination tests indicated that seeds germinated readily in laboratory conditions without stratification; therefore, no conventional wet–cold stratification treatment was applied.

**Table 1.** Family, functional group and seed collection site for all species included in this study. Additional details on habitat, collection date, collectors and collection geographical coordinates are provided in Supplementary Data Table S1. Species names follow the International Plant Names Index (IPNI, 2026).

Family	Species	Functional group	Collection site
Asteraceae	*Taraxacum bracteatum* Dahlst.	Forb	Odindalen, Svalbard
Betulaceae	*Betula nana* L.	Woody plant	Kilpisjärvi Biological Station, Finland
	*Betula pubescens* Ehrh.	Woody plant	Kilpisjärvi Biological Station, Finland
Brassicaceae	*Arabis alpina* L.	Forb	Låktatjåkko Mountain, Abisko, Sweden
	*Cochlearia groenlandica* L.	Forb	Odindalen, Svalbard
Caryophyllaceae	*Cerastium alpinum* L.	Forb	Polish Polar Station Hornsund, Svalbard
	*Silene acaulis* (L.) Jacq.	Woody plant	Endalen, Svalbard
	*Silene involucrata* (Cham. & Schltdl.) Bocquet	Forb	Odindalen, Svalbard
Cyperaceae	*Eriophorum scheuchzeri* Hoppe	Graminoid	Adventdalen, Svalbard
Ericaceae	*Cassiope tetragona* (L.) D.Don	Woody plant	Nuolja Mountain, Abisko, Sweden
	*Kalmia procumbens* (L.) Gift, Kron & P.F.Stevens ex Galasso, Banfi & F.Conti	Woody plant	Kevo Research Station, Finland
	*Phyllodoce caerulea* (L.) Bab.	Woody plant	Kilpisjärvi Biological Station, Finland
Fabaceae	*Hedysarum alpinum* L.	Forb	Churchill Rocket Research Range, Canada
Juncaceae	*Luzula confusa* Lindeb.	Graminoid	Polish Polar Station Hornsund, Svalbard
Onagraceae	*Epilobium angustifolium* L.	Forb	Svanvik, Norway
	*Epilobium anagallidifolium* Lam.	Forb	Låktatjåkko Mountain, Abisko, Sweden
Plantaginaceae	*Veronica alpina* L.	Forb	Latnjajaure, Sweden
Poaceae	*Anthoxanthum odoratum* L.	Graminoid	Nuolja Mountain, Abisko, Sweden
	*Phleum alpinum* L.	Graminoid	Låktatjåkko, Abisko, Sweden
Polygonaceae	*Bistorta vivipara* (L.) Delarbre	Forb	Qeqertarsuaq, Greenland
	*Oxyria digyna* (L.) Hill	Forb	Odindalen, Svalbard
Rosaceae	*Dryas integrifolia* Vahl	Woody plant	Churchill Rocket Research Range, Canada
Saxifragaceae	*Micranthes nivalis* (L.) Small	Forb	Polish Polar Station Hornsund, Svalbard
	*Saxifraga cernua* L.	Forb	Polish Polar Station Hornsund, Svalbard
	*Saxifraga cespitosa* L.	Forb	Odindalen, Svalbard

### Fire-related treatments

Seeds were subjected to four treatments that simulated Arctic fire and post-fire conditions: (1) high heat (110 °C for 4 min); (2) low heat (50 °C for 10 min); (3) smoke water; and (4) a combination of low heat and smoke. For each species and treatment, three replicate Petri dishes containing 10–20 seeds each (depending on seed availability) were used. Untreated seeds served as controls (22 °C).

The two heat treatments were based on temperature data from an experimental Arctic fire conducted in Greenland (Hermesdorf *et al.*, 2022). The high-heat treatment reflects conditions at the soil surface or for seeds still attached to standing vegetation, whereas the low-heat treatment represents temperatures experienced in the soil seed bank (∼2 cm deep). Treatments were applied in a preheated electric oven (Binder GmbH, Germany), with four to five species processed simultaneously. Seeds were placed in preheated glass Petri dishes arranged randomly in the centre of the oven to minimize edge effects. Internal dish temperatures were monitored using a digital thermometer (Huato Electric Co., S220-T8, China). After heating, seeds were transferred to unheated Petri dishes and allowed to cool to room temperature prior to further processing.

For the smoke-exposure treatment, seeds were immersed in an aqueous smoke solution, an effective and convenient substitute for airborne smoke (see Brown, 1993; Dixon *et al.*, 1995). The solution was prepared using biomass from Arctic shrub and herbaceous plants collected in Abisko, Sweden, in August 2024. Three replicates of 20 g of plant material were heated separately in aluminium containers in an oven at 200 ± 1 °C for 30 min, following Jäger *et al.* (1996). After heating, 200 mL of distilled water was added to each replicate and left to steep for 10 min. The resulting extracts were filtered and combined to produce a single smoke solution. Seeds were imbibed in this solution for 24 h at room temperature (∼22 °C). Control seeds were treated with distilled water for the same duration.

The combined low-heat and smoke treatment involved initially applying the low-heat treatment, followed by a 24 h imbibition in smoke water. Preliminary trials showed that the high-heat treatment was lethal to most species; therefore, its interaction with smoke water was not investigated further.

### Germination trials

After fire-related treatments, seeds were placed on a double layer of filter paper soaked in demineralized water in 60 mm Petri dishes. The dishes were incubated in a climate chamber (Buch & Holen, Panasonic, Japan) in conditions previously identified as optimal for germination of Arctic species (25/15 °C and a 12 h–12 h light–dark photoperiod). Dishes were not tightly sealed, and demineralized water was added as required to maintain consistent moisture levels throughout the experiment. The trial lasted 4 weeks. Germination (radicle emergence ≥2 mm) was scored three times per week for the first 3 weeks, and twice during the final week. After each count, germinated seeds were removed to prevent potential allelopathic effects. At the end of the experiment, all non-germinated seeds were cut to assess embryo presence or emptiness, and the initial number of viable seeds was adjusted accordingly.

### Data analysis

All analyses were conducted using R software v.4.4.3 (R Core Team, 2025). Germination counts were used to calculate three indices: (1) final germination percentage (FGP), calculated as:


$$\mathrm{FGP}=\left(\frac{\mathrm{number}\;\mathrm{of}\;\mathrm{germinated}\;\mathrm{seeds}}{\mathrm{total}\;\mathrm{seeds}\;\mathrm{sown}}\right)\times 100;$$

(2) germination speed (GSP), expressed as the germination speed coefficient:


$$\mathrm{GSP}=\left(\frac{\sum_{i=1}^{k}G_{i}}{\sum_{i=1}^{k}G_{i}X_{i}}\right)\times 100,$$

where *G_i_* is the number of seeds germinating at observation *i*, and *X_i_* is the time (in days) from sowing to observation *i*, with higher values indicating faster germination; and (3) germination synchrony (SYN), which measures the temporal clustering of germination events within a replicate and ranges from zero (completely asynchronous germination) to one (all seeds germinating simultaneously), calculated as:


$$\mathrm{SYN}=\frac{\sum C_{n_{i},2}}{N},$$

where $C_{n_{i},2}$ represents the number of seed pairs germinating simultaneously at observation *i*, and *N* is the total number of possible pairs of germinated seeds All indices were calculated using the ‘GerminaR’ package v.2.1.4 (Lozano-Isla *et al.*, 2019). These indices were then used to assess treatment effects at three levels: overall (all species pooled), functional groups (graminoids, forbs and woody species) and species level. Functional-group classification was based on growth form and life-history characteristics (Table 1).

#### High heat

Most species failed to germinate following the high-heat exposure treatment, hence only the final germination percentage was analysed. Germination speed and synchrony were not calculated, because these indices require germination values greater than zero.

To quantify the overall effect of the high-heat treatment, we modelled final germination counts (germinated vs. non-germinated seeds per dish) using a binomial mixed-effects model fitted with the ‘glmmTMB’ package (Brooks *et al.*, 2017). Treatment (control vs. high heat) was included as a fixed effect, and we included random intercepts for species and for replicates nested within species (1|species/replicate) to account for dish-level and interspecific variation. Fixed-effect estimates were evaluated with Wald *z*-tests and back-transformed to obtain germination probabilities.

To test whether functional groups differed in their sensitivity to high-temperature exposure, we extended the model by adding functional group and its interaction with treatment as fixed effects. The significance of the interaction was evaluated using likelihood-ratio (LRT) tests. Functional-group level estimated marginal means and pairwise treatment contrasts were obtained on the response scale using the ‘emmeans’ package (Lenth, 2023).

To estimate species-specific responses for the three species that germinated in the high-heat treatment, we fitted separate binomial mixed-effects models at the seed level. Because only three Petri dishes per species and treatment were available, each seed was treated as an independent observation rather than modelling dish-level counts. Seed counts within each dish were expanded into one row per seed with a binary outcome of ‘germinated’ (1) or ‘not germinated’ (0), and a random intercept for Petri dish ID was included to account for within-dish non-independence. The resulting model [germinated ∼ treatment × species + (1|Petri Dish ID), family = binomial] increased statistical power while appropriately modelling dish-level clustering.

All high-heat models used a logit link and Laplace approximation for estimation. Model fit was assessed using simulation-based diagnostics (i.e. dispersion, zero-inflation, Kolmogorov–Smirnov tests and Q–Q plots) with the ‘DHARMa’ package (Hartig, 2024). No violations of assumptions were detected.

#### Low heat and smoke

To quantify the effects of low heat, smoke and their combination (low heat + smoke) on final germination percentage, we fitted binomial mixed-effects models using the same fixed and random effects structure described above (i.e. treatment as a fixed effect; species and dish nested within species as random effects). Treatment was analysed as a single categorical factor with four levels (control, low-heat, smoke and low heat + smoke). Functional-group differences were evaluated by adding functional group and its interaction with treatment as fixed effects, and species-specific responses were assessed using a model that included treatment and species as fixed factors. Estimated marginal means and treatment contrasts were used to summarize both functional-group and species-specific responses.

Germination speed was analysed using a linear mixed-effects model fitted to log-transformed values with the ‘lme4’ package (Bates *et al.*, 2015). Treatment was included as a fixed effect, and random intercepts for species and for replicates nested within species accounted for interspecific and dish-level variation. Residual diagnostics confirmed that assumptions of normality and homoscedasticity were met. Estimated marginal means were back-transformed to the original scale, and Tukey-adjusted pairwise comparisons were used to test for treatment differences relative to the control. Functional-group- and species-specific patterns were examined using models that included the relevant interaction terms (functional group × treatment or species × treatment) as described above.

Germination synchrony values are bounded between zero and one and can include zeros; therefore, we used a Tweedie mixed-effects model with a log link in the ‘glmmTMB’ package (Brooks *et al.*, 2017) to analyse the data. For the overall model, treatment was included as a fixed effect, and replicates nested within species were included as random intercepts. Estimated marginal means (on the response scale) were back-transformed to the original zero–one scale, and Tukey-adjusted pairwise contrasts were used to compare synchrony among treatments. As above, functional-group- and species-specific responses were obtained from models that included the relevant interaction terms.

All models were checked using simulation-based diagnostics, and no violations of model assumptions were detected.

## RESULTS

### High heat

#### Final germination percentage

Exposure to high heat caused a pronounced reduction in final germination percentage, dropping from 78.4 % in control conditions to 2.1 % (*P* < 0.001; Fig. 1A). This represents extreme sensitivity to high temperatures across species (logit estimate = −5.14 ± 0.27, *z* = −19.3).

**Fig. 1.**
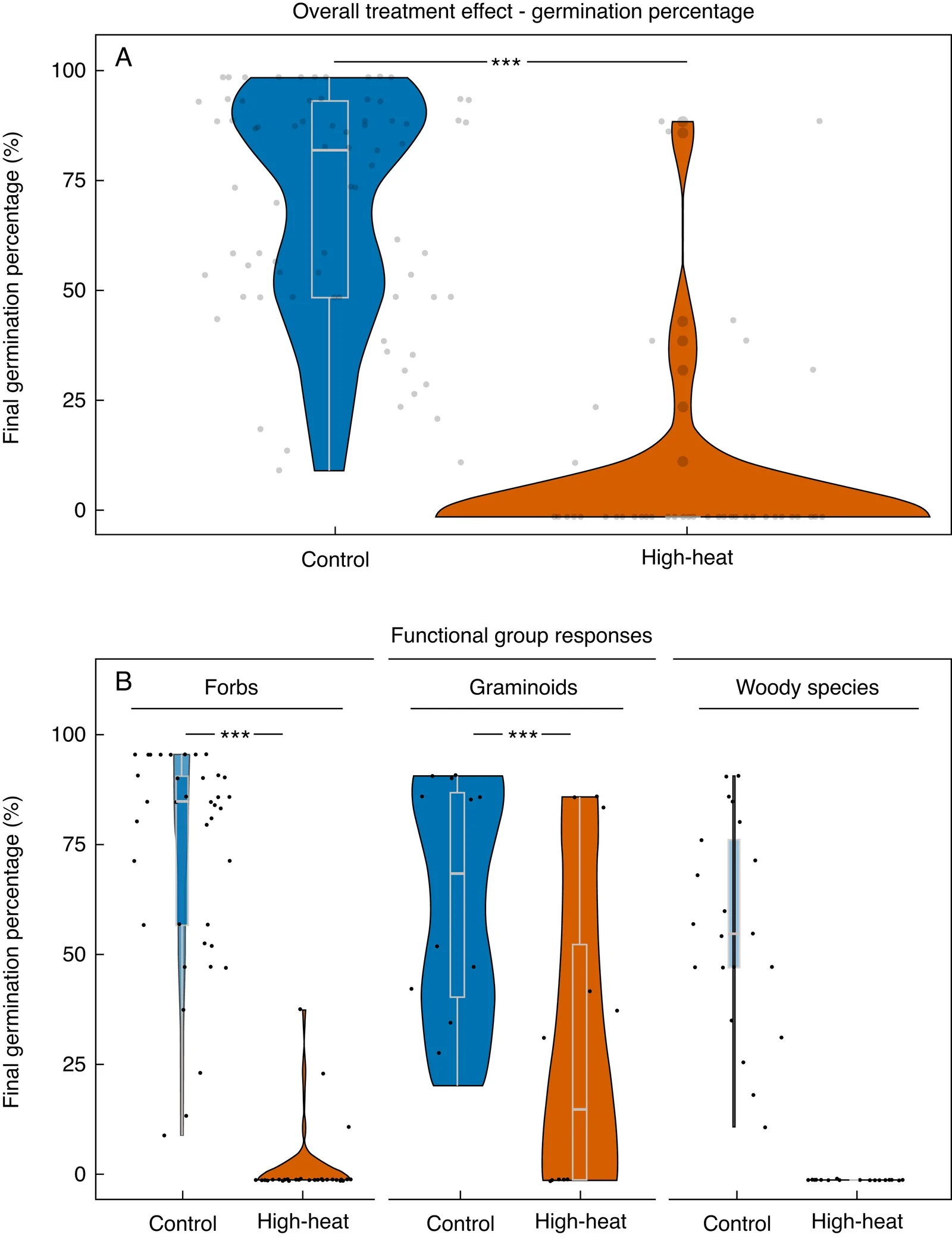
Effects of high-heat treatment on the final germination percentage across 25 Arctic vascular plant species. (A) Overall reduction in germination following high-heat exposure. (B) Responses by functional group. In this and subsequent figures, violin plots depict the distribution (kernel density) of dish-level germination values; embedded box-and-whisker plots show the median and interquartile range; and points represent individual Petri dishes. ***P < 0.001.

Functional groups differed in their responses (treatment × group interaction: LRT = 80.3, *P* < 0.001; Fig. 1B). Forbs exhibited near-complete germination failure, with only 1 of 14 species germinating (odds ratio = 0.001, *P* < 0.001). Graminoids were less affected, with two of four species showing any germination (odds ratio = 0.12, *P* < 0.001). Woody species did not germinate under high-heat exposure; although model contrasts could not be estimated owing to complete separation, raw germination percentages confirm zero germination.

Only three species germinated following high-heat exposure, and their responses were contrasting (Supplementary Data Table S2; Fig. S2). *Epilobium angustifolium* L. declined from 94.7 to 26.9 % (odds ratio = 0.02, *P* < 0.001); *Phleum alpinum* L. showed no detectable change (92.9 vs. 89.3 %; odds ratio = 0.64, *P* = 0.58); and *Anthoxanthum odoratum* L. exhibited a small, non-significant increase (29.8 vs. 39.3 %; odds ratio = 1.52, *P* = 0.38).

### Low heat and smoke

#### Final germination percentage

Low heat, smoke and their combination did not significantly affect overall germination proportions (Fig. 2A). Germination remained high in all treatments (76–80 %), and none differed from the control: low heat (model estimate = −0.08 ± 0.10, *P* = 0.43; 76 % predicted germination), smoke (+0.13 ± 0.10, *P* = 0.20; 80 %) or low-heat + smoke (+0.10 ± 0.10, *P* = 0.34; 79 %). Pairwise contrasts confirmed the absence of treatment effects (all *P* > 0.40).

**Fig. 2.**
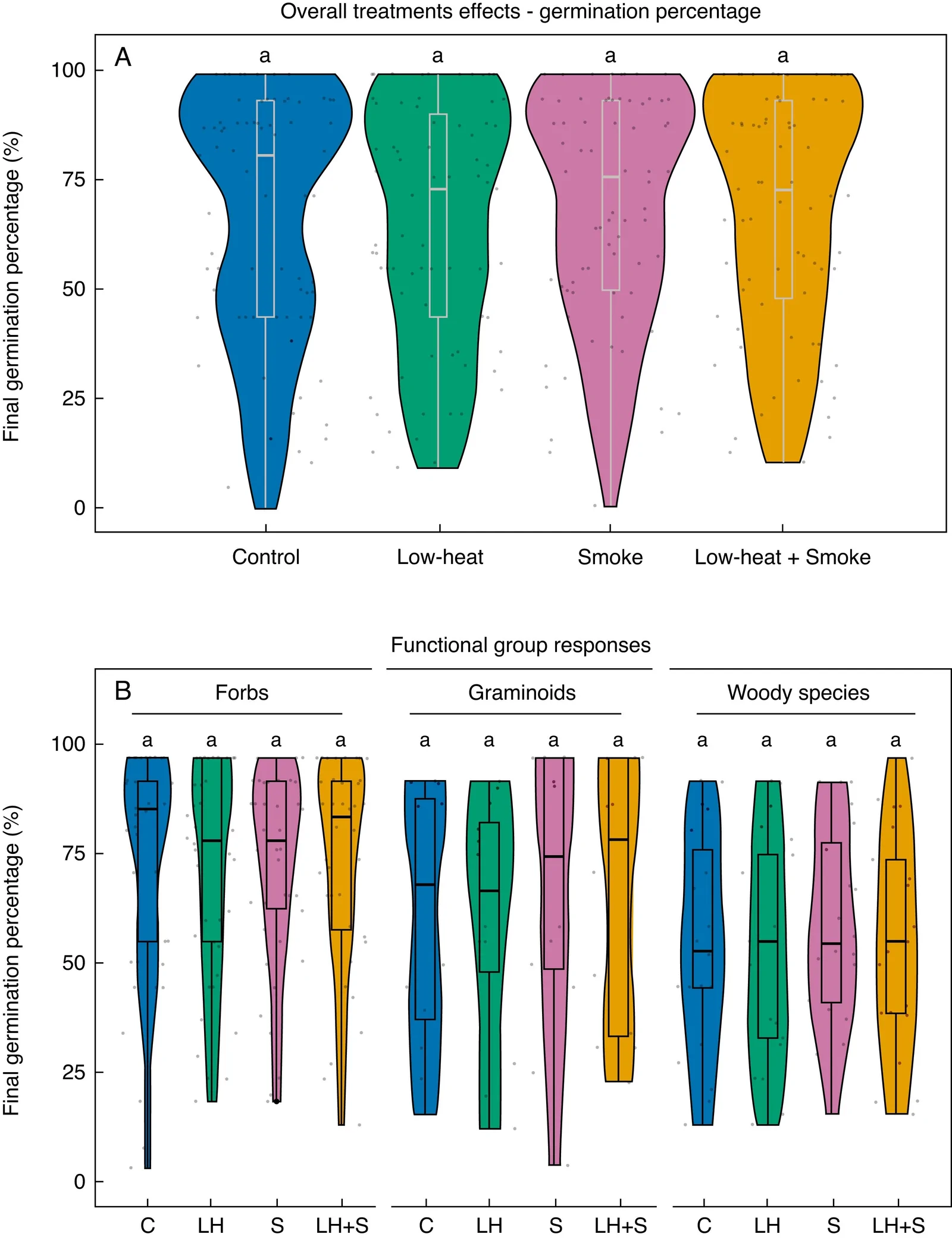
Effects of low-heat and smoke treatments on germination across 25 Arctic vascular plant species. (A) Final germination percentage under low-heat (LH), smoke (S) and combined low-heat + smoke (LH + S) treatments. (B) Functional group responses showing broadly similar patterns across forbs, graminoids and woody species. Different letters indicate statistically significant differences among treatment groups.

Functional groups showed similarly consistent responses, with no evidence of a treatment × group interaction (LRT = 5.07, d.f. = 6, *P* = 0.53). Forbs maintained high germination (82.9–85.1 %); graminoids ranged from 73.7 to 80.4 % and woody species from 59.2 to 67.8 % (Fig. 2B).

At the species level, only 1 of the 75 contrasts tested (i.e. three treatments vs. control per species) was statistically significant: *Hedysarum alpinum* L. showed higher germination in the low-heat + smoke treatment than in the control (odds ratio = 3.18, 95 % confidence interval: 1.14–8.87, *P* = 0.022). No other species displayed evidence of treatment-related changes in final germination (Supplementary Data Table S2; Fig. S3).

#### Germination speed

Smoke treatments modestly but consistently accelerated germination, whereas low heat alone had no effect (Fig. 3A). Back-transformed estimates were similar in the control and low-heat treatments (12.6 vs. 12.0; *P* = 0.18), but higher in smoke (13.8; *P* = 0.004) and low heat + smoke (14.0; *P* < 0.001).

**Fig. 3.**
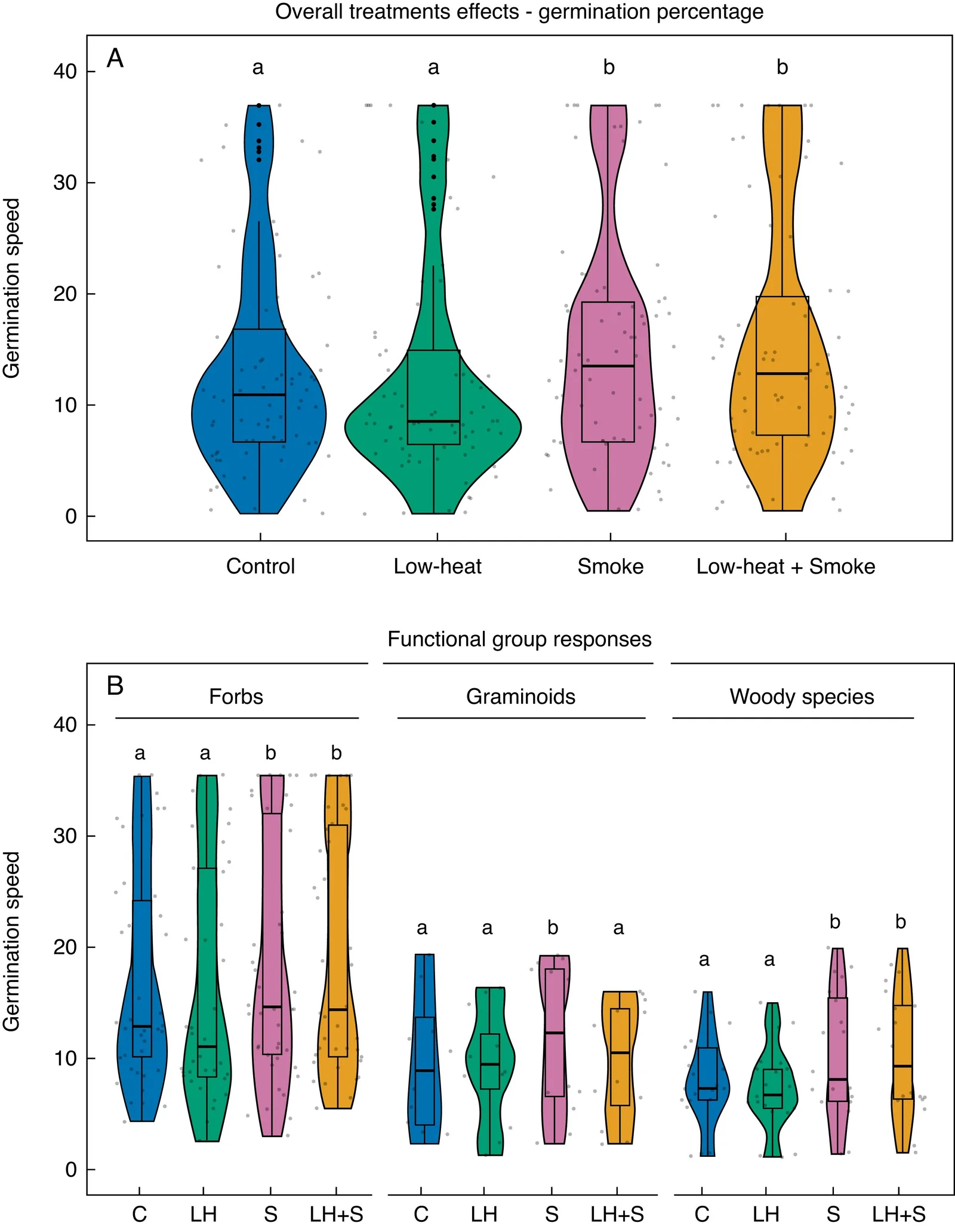
Effects of treatments on germination speed across 25 Arctic vascular plant species. (A) Overall germination speed under low-heat (LH), smoke (S) and combined low-heat + smoke (LH + S). (B) Functional group responses showing similar patterns across forbs, graminoids and woody species. Values are back-transformed means (±95 % confidence intervals) from linear mixed models. Different letters indicate statistically significant differences among treatment groups.

Different functional groups responded similarly to the treatments (treatment × group interaction: *F* = 1.16, *P* = 0.33; Fig. 3B). Across all groups, low heat did not influence germination speed, whereas smoke and low heat + smoke generally increased it. Forbs [Δlog(GSP) = 0.10, *P* = 0.015] and woody species [Δlog(GSP) = 0.14, *P* = 0.024] exhibited the strongest acceleration under low heat + smoke, whereas graminoids showed only weak, non-significant trends in the same direction. Back-transformed means indicated modest increases in germination speed in smoke treatments across all groups.

Responses of germination speed to the treatments (low heat, smoke and their combination) varied across species (Supplementary Data Fig. S4) and did not cluster by functional group. Ten of the 25 species showed at least one significant contrast (17 of 75 contrasts, *P* < 0.05). Low heat slowed germination in five taxa: *Arabis alpina* L., *Dryas integrifolia* Vahl, *Micranthes nivalis* (L.) Small, *Phleum alpinum* L. and *Saxifraga cespitosa* L. [Δlog(GSP) ≈ −0.12 to −0.29, i.e. GSP ≈ 10–25 % slower; Supplementary Data Table S3]. In contrast, smoke, either on its own or combined with low heat, commonly accelerated germination in seven species, including *Betula nana* L., *Eriophorum scheuchzeri* Hoppe, *Luzula confusa* Lindeb., *Silene involucrata* (Cham. & Schltdl.) Bocquet and *Taraxacum bracteatum* Dahlst. [Δlog(GSP) ≈ 0.18–0.70, i.e. GSP ≈ 20–100 % faster; Supplementary Data Table S3].

#### Germination synchrony

Overall germination synchrony was not affected by any of the experimental treatments (low heat, smoke and their combination). Mixed-effects models detected no effect of low heat, smoke or their combination (all *P* > 0.32). Estimated synchrony values were similar across treatments, ranging from 0.40 in the control to 0.43 in the smoke treatment. Pairwise comparisons and Dunnett’s tests confirmed that none of the treatments differed significantly from the control (all *P* > 0.61; Fig. 4A).

**Fig. 4.**
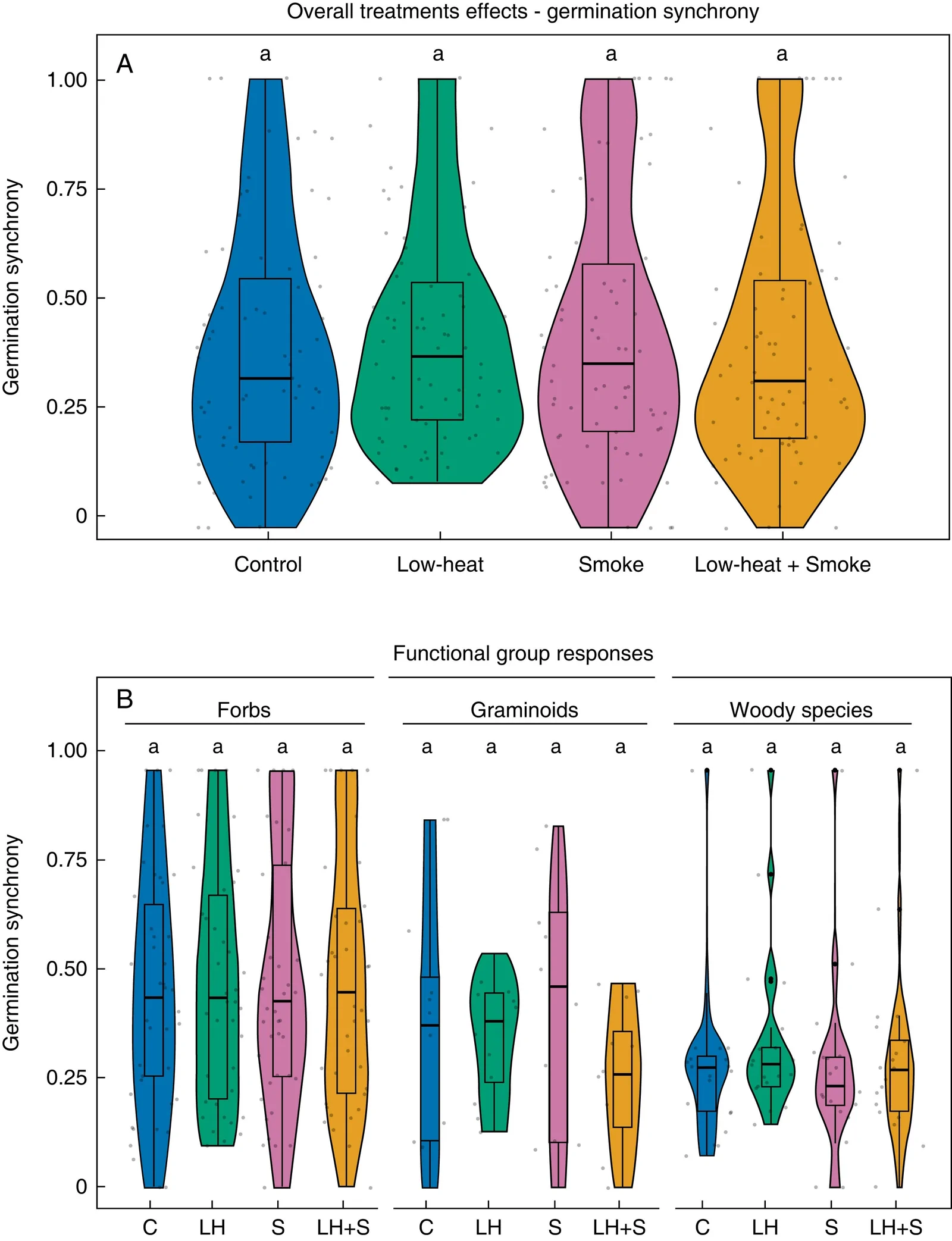
Effects of experimental treatments on germination synchrony across 25 Arctic vascular plant species. (A) Germination synchrony under low-heat, smoke and combined low-heat + smoke treatments. (B) Estimated marginal means (±95 % confidence intervals) for forbs, graminoids and woody species.

Functional groups showed the same pattern. The treatment × functional group interaction was non-significant (LRT: *F*_6_,_215_._63_ = 1.28, *P* = 0.27; Fig. 4B), indicating that synchrony responses did not differ among forbs, graminoids and woody species. Dunnett-adjusted contrasts also showed no significant deviations from the control in any group (all *P* > 0.26).

In contrast, species-level responses were more variable. Nine of the 25 species showed at least one significant difference from the control (Supplementary Data Table S4; Fig. S5). Synchrony increased under smoke exposure in seven species [*Bistorta vivipara* (L.) Delarbre, *Epilobium angustifolium* L., *Eriophorum scheuchzeri* Hoppe, *Luzula confusa* Lindeb., *Saxifraga cespitosa* L., *Silene involucrata* Cham. & Schltdl. Bocquet and *Taraxacum bracteatum* Dahlst] and decreased in response to low heat in two species (*Arabis alpina* L. and *Phleum alpinum* L.). These species-specific effects indicate that low heating and smoke can alter germination synchrony in some taxa even when community-wide averages remain unchanged.

## DISCUSSION

This study provides a multi-species experimental assessment of how seeds of Arctic species respond to fire-related cues. High-heat exposure caused near-complete germination failure across species, whereas low-heat exposure had little effect on overall germination but did alter germination speed and synchrony in some taxa. Smoke water exposure accelerated overall germination, but without affecting final germination proportion. Together, these results suggest that seeds of the studied Arctic species exhibit limited fire-adaptive traits: they are highly sensitive to high temperatures and show no increase in germination in response to low heat or smoke cues.

### Fire heat acts as a strong ecological filter on Arctic seeds

Our results show that fire-induced temperatures at or near the soil surface, reflected by our high-heat treatment, are lethal for almost all studied species. This lack of physical or physiological tolerance is not surprising from an ecological and evolutionary perspective, because fire has played only a minor role in the recent history of Arctic ecosystems (Masrur *et al.*, 2017; Descals *et al.*, 2022; Gosden *et al.*, 2022) and has therefore not acted as a selective force shaping seed traits. In contrast, in fire-adapted regions, such as temperate ecosystems of Australia (Hodges *et al.*, 2021; Tangney *et al.*, 2025) or tropical grasslands in Brazil (Paredes *et al.*, 2018; Ramos *et al.*, 2019), heat levels comparable to our high-heat treatment (∼110 °C) are generally not lethal to seeds.

Graminoids were less negatively affected than forbs or woody species, a pattern that aligns with graminoid-dominated post-fire trajectories in the Arctic (Heim *et al.*, 2025) and suggests that their seed-level heat tolerance might complement their well-known capacity for resprouting (Bret-Harte *et al.*, 2013; Hollingsworth *et al.*, 2021). Nevertheless, this interpretation requires caution, because it is based on a limited number of graminoid species (*n* = 4), and expanded sampling across taxonomic, intraspecific and regional diversity is needed to assess whether this pattern is consistent.

Only three species germinated following high-heat exposure. *Epilobium angustifolium* L. and *Phleum alpinum* L. are well-known pioneers of fire-affected boreal and tundra sites (Bret-Harte *et al.*, 2013; Racine *et al.*, 2004, 1987), whereas *Anthoxanthum odoratum* L. is a dominant invasive in post-fire alpine grasslands in Australia (Verrall and Pickering, 2019). Their tolerance might reflect phylogenetic heritage rather than local adaptation, because they belong to lineages with fire-responsive traits (e.g. Poales; Lamont *et al.*, 2019). However, this explanation is not consistent across all taxa, because some study species that are members of typically fire-adapted families, such as Ericaceae or Fabaceae (Lamont *et al.*, 2019), failed to germinate, despite the fact that close relatives have shown fire-related responses in European heathlands (Bargmann *et al.*, 2014) and boreal forests (Granström and Schimmel, 1993).

In contrast to high heat, the low-heat treatment, representing subsurface temperatures during a fire, was non-lethal for the study species and produced only modest changes in germination traits. These results align with findings from both fire-adapted ecosystems (e.g. boreal forest: Granström and Schimmel, 1993; temperate grasslands in Australia: Hodges *et al.*, 2021; Mediterranean shrublands in South Africa: Hall *et al.*, 2017) and non-fire-adapted ecosystems (e.g. tropical wetlands: Soares *et al.*, 2021), where moderate heating (∼60 °C) is generally not lethal to seeds. However, unlike such fire-prone floras, where comparable temperatures often stimulate germination by breaking physical dormancy, we detected no increase in germination proportion. Instead, some species germinated more slowly or less synchronously, suggesting an absence of heat-responsive dormancy mechanisms, which reinforces the hypothesis that fire has not shaped the germination traits of Arctic plants.

Although overall responses to low-heat exposure were modest, several species exhibited consistent shifts in germination behaviour. For example, *Arabis alpina* L., *Micranthes nivalis* (L.) Small and *Phleum alpinum* L. germinated more slowly and less synchronously following low-heat exposure, whereas *Luzula confusa* Lindeb. showed the opposite pattern, with faster and more synchronous germination. *Saxifraga cespitosa* L. exhibited a mixed response, with reduced germination speed but increased synchrony. These contrasting species-specific responses suggest that moderate heating might influence the timing of germination in some Arctic species even when the final germination proportion remains unchanged. Notably, we found no evidence that low-heat exposure differentially affected functional groups, suggesting that the contrasting graminoid- and shrub-dominated post-fire trajectories reported across Arctic ecosystems (Heim *et al.*, 2025) are unlikely to arise from differences in the responses of buried seeds to fire. Instead, these trajectories might reflect species-specific responses, variation in seed survival at the soil surface, vegetative regeneration, seed dispersal and post-fire establishment.

From an ecological perspective, Arctic fires are likely to kill surface seeds, whereas those buried in the soil may survive. Fire might therefore act as a strong ecological filter, favouring species with traits that enhance seed burial and promote the formation of permanent soil seed banks (e.g. small and spherical seeds; see Wang *et al.*, 2024), while disadvantaging species with transient or aerial seed banks. This filtering effect is likely to be amplified by the interaction between site moisture, soil fertility and the depth of the organic layer throughout the Arctic. Dry tundra sites, for example, typically have thinner organic soil layers that can burn more intensely and uniformly, thereby increasing the probability of seed bank mortality. Seed dispersal phenology will also shape vulnerability to fire, with late-dispersing species (i.e. those releasing seeds in autumn or winter) at greater risk because fires are projected to occur more frequently in late summer and autumn (French *et al.*, 2015).

### Smoke cues might facilitate post-fire recruitment

The accelerated germination observed after exposure to smoke water for many of the species shows that Arctic vascular plants can respond to smoke-derived chemical cues despite the historical rarity of fire in the region. The lack of change in final germination percentage, a common effect of smoke in fire-prone environments such as European heathlands (Bargmann *et al.*, 2014) or Australian grasslands (Hodges *et al.*, 2021), suggests that smoke compounds (e.g. butenolide) might have stimulated metabolic activity or relaxed residual physiological constraints on germination, rather than breaking fire-related dormancy (Staden *et al.*, 2000; Light *et al.*, 2004, 2009). This indicates a non-evolutionary response to smoke, similar to effects reported for species in other low-fire-frequency environments, such as arid zones (Merritt *et al.*, 2006), alpine herbfields (Vázquez-Ramírez and Venn, 2025) or wet rainforest (Ferraz *et al.*, 2013).

From an ecological perspective, faster germination following smoke exposure could enhance post-fire recruitment by allowing seedlings to establish earlier, before they are outcompeted by resprouting plants. Such rapid establishment might be particularly advantageous where fire has removed moss and litter layers, exposing mineral soil, increasing soil temperature and temporarily enhancing nutrient availability (Racine *et al.*, 2004, 1987). Faster germination would give seedlings a competitive advantage, because it would enable them to accumulate sufficient biomass before winter begins, thereby increasing their chances of survival (Milbau *et al.*, 2009). However, the ecological benefit of accelerated germination ultimately depends on the availability of viable seeds after the fire (i.e. whether enough seeds survived the fire heat shock) and the presence of suitable microsites for establishment (Graae *et al.*, 2011).

In this context, although the overall pattern indicates that smoke generally accelerates germination across taxa and functional groups, some modest species-level differences were found (e.g. Supplementary Data Table S3; Fig. S4). Smoke-related cues produced the strongest phenological shifts in a subset of early successional forbs and sedges (e.g. *Eriophorum scheuchzeri* Hoppe, *Silene involucrata* Cham. & Schltdl., Bocquet and *Taraxacum bracteatum* Dahlst.), whereas species that typically occur on dry, wind-exposed ridges and fellfields (e.g. *Micranthes nivalis* L. Small, *Saxifraga cespitosa* L. and *Cerastium alpinum* L.) showed little or no response. These differences might influence which species are able to capitalize on the brief post-fire recruitment window, contributing to shifts in post-fire vegetation composition.

From a management perspective, the stimulatory effect of smoke water on germination speed (and synchrony in some species) could be used to pretreat seeds prior to seed-based restoration activities in the Arctic, thereby potentially enhancing the success of these efforts.

### Study limitations and future directions

Our results provide insights into the potential impact of fire on Arctic seed-based regeneration, thus our findings can be generalized to similar ecosystems and taxa. Nonetheless, it is always important to consider limitations, and we note that the following aspects could be extended in future study designs. First, our study focused on 25 widespread species; we sampled only one population per species, and we did not account for potential effects of fire history. Seed stress tolerance can vary among populations, and seeds from previously fire-affected sites might differ in their sensitivity to heat (Zaki *et al.*, 2021) and smoke (Manela *et al.*, 2019). To understand better how fire influences Arctic seeds, future studies should broaden the taxonomic scope to rare species and include multiple populations along a fire-history gradient, thereby enabling tests of potential local adaptation or transgenerational plasticity in seed responses to heat and smoke.

Second, we used a single concentration of smoke water. Seed responses to smoke vary depending on the concentration and exposure time and can shift from stimulatory to inhibitory (see Light *et al.*, 2004; Merritt *et al.*, 2006). Future studies should therefore test a broader range of concentrations and exposure durations. Furthermore, our smoke solution was produced from a mixture of Arctic plant biomass, and because smoke chemistry differs depending on the type of fuel, smoke generated from different types of vegetation (e.g. graminoid-dominated or shrub-dominated tundra) might result in different germination responses (Jäger *et al.*, 1996). Future studies should aim to produce smoke from single-species fuels or community-specific fuel to test how biomass composition influences smoke chemistry and seed responses.

Finally, to our knowledge, there is no published information on the temperatures reached during Arctic fires (although we did not conduct a systematic literature review), representing an important knowledge gap that must be addressed to understand better how seeds respond to fire-related heat. Although our heat treatments were based on *in situ* measurements from an Arctic experimental burn (Hermesdorf *et al.*, 2022), the large temperature variation between the low- and high-heat treatments prevented us from determining lethal temperature thresholds for our study species. Future work should therefore record temperature profiles during real fire events to inform experimental designs more effectively and also expose seeds to continuous temperature and heating duration gradients to determine their lethal thresholds (see the pyro-niche framework proposed by Tangney *et al.*, 2025). Such experiments would also allow comparisons with other high-latitude systems, such as boreal forests, where fire is an important ecological driver.

### Conclusion

We provide a multi-species experimental assessment of fire tolerance in Arctic seeds. We found that 88 % of species were unable to withstand high temperatures, and neither moderate heat nor smoke-related cues increased the overall germination proportion. Our findings suggest that fire-related heat exposure acts as an ecological filter in the Arctic: high surface temperatures kill seeds, whereas elevated subsurface temperatures have little effect on seed viability and germination. Consequently, species with traits that promote seed burial and the formation of soil seed banks might be better positioned to persist under fire regimes. Smoke accelerated overall germination, suggesting that surviving seeds might be able to take advantage of short recruitment windows following fire. Given that fire frequency, intensity, severity and extent increase owing to rapid Arctic warming, seed-based regeneration is likely to play an important role in vegetation dynamics. Understanding how these processes scale across landscapes and interact with changing fire regimes is essential for predicting the resilience of Arctic ecosystems and for informing seed-based restoration in a rapidly transforming Arctic.

## Supplementary Material

mcag236_Supplementary_Data

## Data Availability

Data and analyses, diagnostics and figure generation were scripted to ensure reproducibility, and full code is archived and available at https://doi.org/10.5281/zenodo.18302947.

## SUPPLEMENTARY DATA

Supplementary data are available at *Annals of Botany* online and consist of the following. Table S1: taxonomic identity, functional group, habitat and collection metadata of the studied species. Table S2: pairwise contrasts comparing the effects of low-heat, smoke and combined low-heat + smoke treatments against the control on final germination percentage. Table S3: pairwise contrasts comparing the effects of low-heat, smoke and combined low-heat + smoke treatments against the control on final germination speed. Table S4: pairwise contrasts comparing the effects of low-heat, smoke and combined low-heat + smoke treatments against the control on final germination synchrony. Figure S1: seed collection sites for all species included in the study. Figure S2: final germination percentage for each species in control and high-temperature treatments. Figure S3: species-specific effects of low-heat, smoke and their combination on final germination percentage. Figure S4: species-specific effects of low-heat, smoke and their combination on germination speed. Figure S5: species-specific effects of low-heat, smoke and their combination on germination synchrony.

## AI assistance

No AI assistance to declare.

## References

[mcag236-B1] Bargmann T , Måren IE, Vandvik V. 2014. Life after fire: smoke and ash as germination cues in ericads, herbs and graminoids of northern heathlands. Applied Vegetation Science 17: 670–679. doi:10.1111/avsc.12106

[mcag236-B2] Bates D , Mächler M, Bolker B, Walker S. 2015. Fitting linear mixed-effects models using lme4. Journal of Statistical Software 67: 1–48. doi:10.18637/jss.v067.i01

[mcag236-B3] Bret-Harte MS , Mack MC, Shaver GR, et al 2013. The response of Arctic vegetation and soils following an unusually severe tundra fire. Philosophical Transactions of the Royal Society B: Biological Sciences 368: 20120490. doi:10.1098/rstb.2012.0490 PMC3720061 23836794

[mcag236-B4] Brooks ME , Kristensen K, Benthem KJV, et al 2017. glmmTMB balances speed and flexibility among packages for zero-inflated generalized linear mixed modeling. The R Journal 9: 378. doi:10.32614/RJ-2017-066

[mcag236-B5] Brown NAC . 1993. Promotion of germination of fynbos seeds by plant-derived smoke. The New Phytologist 123: 575–583. doi:10.1111/j.1469-8137.1993.tb03770.x 33874107

[mcag236-B6] Chen Y , Hu FS, Lara MJ. 2021. Divergent shrub-cover responses driven by climate, wildfire, and permafrost interactions in Arctic tundra ecosystems. Global Change Biology 27: 652–663. doi:10.1111/gcb.15451 33216446

[mcag236-B7] Descals A , Gaveau DLA, Verger A, Sheil D, Naito D, Peñuelas J. 2022. Unprecedented fire activity above the Arctic circle linked to rising temperatures. Science 378: 532–537. doi:10.1126/science.abn9768 36378957

[mcag236-B8] Dixon KW , Roche S, Pate JS. 1995. The promotive effect of smoke derived from burnt native vegetation on seed germination of Western Australian plants. Oecologia 101: 185–192. doi:10.1007/BF00317282 28306789

[mcag236-B9] Ferraz IDK , Arruda YMBC, Van Staden J. 2013. Smoke-water effect on the germination of Amazonian tree species. South African Journal of Botany 87: 122–128. doi:10.1016/j.sajb.2013.04.004

[mcag236-B10] French NHF , Jenkins LK, Loboda TV, et al 2015. Fire in Arctic tundra of Alaska: past fire activity, future fire potential, and significance for land management and ecology. International Journal of Wildland Fire 24: 1045–1061. doi:10.1071/WF14167

[mcag236-B11] Frost GV , Loehman RA, Saperstein LB, et al 2020. Multi-decadal patterns of vegetation succession after tundra fire on the Yukon-Kuskokwim Delta, Alaska. Environmental Research Letters 15: 025003. doi:10.1088/1748-9326/ab5f49

[mcag236-B12] Gaglioti B , Berner L, Jones B, et al 2021. Tussocks enduring or shrubs greening: alternate responses to changing fire regimes in the Noatak River Valley, Alaska. Journal of Geophysical Research: Biogeosciences 126: e2020JG006009. doi:10.1029/2020JG006009

[mcag236-B13] Gosden B , Lovell H, Hardiman M. 2022. Wildfire incidence in western Kalaallit Nunaat (Greenland) from 1995 to 2020. International Journal of Wildland Fire 31: 1033–1042. doi:10.1071/WF22063

[mcag236-B14] Graae BJ , Ejrnæs R, Lang SI, Meineri E, Ibarra PT, Bruun HH. 2011. Strong microsite control of seedling recruitment in tundra. Oecologia 166: 565–576. doi:10.1007/s00442-010-1878-8 PMC3094527 21170749

[mcag236-B15] Granström A , Schimmel J. 1993. Heat effects on seeds and rhizomes of a selection of boreal forest plants and potential reaction to fire. Oecologia 94: 307–313. doi:10.1007/BF00317103 28313665

[mcag236-B16] Hall SA , Newton RJ, Holmes PM, Gaertner M, Esler KJ. 2017. Heat and smoke pre-treatment of seeds to improve restoration of an endangered Mediterranean climate vegetation type. Austral Ecology 42: 354–366. doi:10.1111/aec.12449

[mcag236-B17] Hartig F . 2024. DHARMa: Residual Diagnostics for Hierarchical (Multi-Level/Mixed) Regression Models. R package version 0.2.0. https://CRAN.R-project.org/package=DHARMa.

[mcag236-B18] He J , Loboda T, Chen D, French N. 2022. Cloud-to-ground lightning and near-surface fire weather control wildfire occurrence in Arctic tundra. Geophysical Research Letters 49: e2021GL096814. doi:10.1029/2021GL096814

[mcag236-B19] Heim RJ , Rocha AV, Zemlianskii V, et al 2025. Arctic tundra ecosystems under fire—alternative ecosystem states in a changing climate? The Journal of Ecology 113: 1042–1056. doi:10.1111/1365-2745.70022

[mcag236-B20] Hermesdorf L , Elberling B, D’Imperio L, Xu W, Lambæk A, Ambus PL. 2022. Effects of fire on CO_2_, CH_4_, and N_2_O exchange in a well-drained Arctic heath ecosystem. Global Change Biology 28: 4882–4899. doi:10.1111/gcb.16222 PMC9544550 35543023

[mcag236-B21] Hodges JA , Price JN, Nicotra AB, Neeman T, Guja LK. 2021. Smoke and heat accelerate and increase germination in fire-prone temperate grassy ecosystems. Ecosphere 12: e03851. doi:10.1002/ecs2.3851

[mcag236-B22] Hollingsworth TN , Breen AL, Hewitt RE, Mack MC. 2021. Does fire always accelerate shrub expansion in Arctic tundra? Examining a novel grass-dominated successional trajectory on the Seward Peninsula. Arctic, Antarctic, and Alpine Research 53: 93–109. doi:10.1080/15230430.2021.1899562

[mcag236-B23] IPNI . 2026. International Plant Names Index. Published on the Internet http://www.ipni.org, The Royal Botanic Gardens, Kew, Harvard University Herbaria & Libraries and Australian National Herbarium (8 August 2026, date last accessed).

[mcag236-B24] Jäger AK , Light ME, Van Staden J. 1996. Effects of source of plant material and temperature on the production of smoke extracts that promote germination of light-sensitive lettuce seeds. Environmental and Experimental Botany 36: 421–429. doi:10.1016/S0098-8472(96)01024-6

[mcag236-B25] Jones BM , Breen AL, Gaglioti BV, et al 2013. Identification of unrecognized tundra fire events on the north slope of Alaska. Journal of Geophysical Research: Biogeosciences 118: 1334–1344. doi:10.1002/jgrg.20113

[mcag236-B26] Kasischke ES , Turetsky MR. 2006. Recent changes in the fire regime across the North American boreal region—spatial and temporal patterns of burning across Canada and Alaska. Geophysical Research Letters 33. doi:10.1029/2006GL025677

[mcag236-B27] Lamont BB , He T, Yan Z. 2019. Evolutionary history of fire-stimulated resprouting, flowering, seed release and germination. Biological Reviews of the Cambridge Philosophical Society 94: 903–928. doi:10.1111/brv.12483 30484944

[mcag236-B28] Landhausser SM , Wein RW. 1993. Postfire vegetation recovery and tree establishment at the Arctic treeline: climate-change–vegetation-response hypotheses. The Journal of Ecology 81: 665–672. doi:10.2307/2261664

[mcag236-B29] Lenth R . 2023. emmeans: estimated marginal means, aka least-squares means. R package version 1.10.5. https://github.com/rvlenth/emmeans/WorldCat.

[mcag236-B30] Light ME , Daws MI, Van Staden J. 2009. Smoke-derived butenolide: towards understanding its biological effects. South African Journal of Botany 75: 1–7. doi:10.1016/j.sajb.2008.10.004

[mcag236-B31] Light ME , van Staden J, Bornman CH. 2004. The potential of smoke in seed technology. South African Journal of Botany 70: 97–101. doi:10.1016/S0254-6299(15)30311-2

[mcag236-B32] Liu Y , Riley WJ, Keenan TF, et al 2022. Dispersal and fire limit Arctic shrub expansion. Nature Communications 13: 3843. doi:10.1038/s41467-022-31597-6 PMC9253140 35788612

[mcag236-B33] Lozano-Isla F , Benites-Alfaro OE, Pompelli MF. 2019. GerminaR: an R package for germination analysis with the interactive web application “GerminaQuant for R”. Ecological Research 34: 339–346. doi:10.1111/1440-1703.1275

[mcag236-B34] Mack MC , Bret-Harte MS, Hollingsworth TN, et al 2011. Carbon loss from an unprecedented Arctic tundra wildfire. Nature 475: 489–492. doi:10.1038/nature10283 21796209

[mcag236-B35] Manela N , Dagon E, Semesh H, Ovadia O. 2019. Smoke interacts with fire history to stimulate soil seed bank germination in Mediterranean woodlands. Journal of Plant Ecology 12: 419–427. doi:10.1093/jpe/rty052

[mcag236-B36] Masrur A , Petrov AN, DeGroote J. 2017. Circumpolar spatio-temporal patterns and contributing climatic factors of wildfire activity in the Arctic tundra from 2001–2015. Environmental Research Letters 13: 014019. doi:10.1088/1748-9326/aa9a76

[mcag236-B37] McCarty JL , Aalto J, Paunu V-V, et al 2021. Reviews and syntheses: arctic fire regimes and emissions in the 21st century. Biogeosciences 18: 5053–5083. doi:10.5194/bg-18-5053-2021

[mcag236-B38] Merritt DJ , Kristiansen M, Flematti GR, et al 2006. Effects of a butenolide present in smoke on light-mediated germination of Australian Asteraceae. Seed Science Research 16: 29–35. doi:10.1079/SSR2005232

[mcag236-B39] Milbau A , Graae BJ, Shevtsova A, Nijs I. 2009. Effects of a warmer climate on seed germination in the subarctic. Annals of Botany 104: 287–296. doi:10.1093/aob/mcp117 PMC2710907 19443459

[mcag236-B40] Paredes MVF , da Cunha ALN, Musso C, Aires SS, Sato MN, Miranda HS. 2018. Germination responses of native and invasive Cerrado grasses to simulated fire temperatures. Plant Ecology & Diversity 11: 193–203. doi:10.1080/17550874.2018.1471626

[mcag236-B41] Racine C , Jandt R, Meyers C, Dennis J. 2004. Tundra fire and vegetation change along a hillslope on the Seward Peninsula, Alaska, U.S.A. Arctic, Antarctic, and Alpine Research 36: 1–10. doi:10.1657/1523-0430(2004)036[0001:TFAVCA]2.0.CO;2

[mcag236-B42] Racine CH , Johnson LA, Viereck LA. 1987. Patterns of vegetation recovery after tundra fires in Northwestern Alaska, U.S.A. Arctic and Alpine Research 19: 461–469. doi:10.1080/00040851.1987.12002628

[mcag236-B43] Ramos DM , Valls JFM, Borghetti F, Ooi MKJ. 2019. Fire cues trigger germination and stimulate seedling growth of grass species from Brazilian savannas. American Journal of Botany 106: 1190–1201. doi:10.1002/ajb2.1345 31449672

[mcag236-B44] Rantanen M , Karpechko AY, Lipponen A, et al 2022. The Arctic has warmed nearly four times faster than the globe since 1979. Communications Earth & Environment 3: 1–10. doi:10.1038/s43247-022-00498-3

[mcag236-B45] R Core Team . R: a language and environment for statistical computing. 2025.Vienna: R Foundation for Statistical Computing.

[mcag236-B46] Soares VC , Scremin-Dias E, Daibes LF, Damasceno-Junior GA, Pott A, de Lima LB. 2021. Fire has little to no effect on the enhancement of germination, but buried seeds may survive in a Neotropical wetland. Flora 278: 151801. doi:10.1016/j.flora.2021.151801

[mcag236-B47] Staden JV , Brown NAC, Jäger AK, Johnson TA. 2000. Smoke as a germination cue. Plant Species Biology 15: 167–178. doi:10.1046/j.1442-1984.2000.00037.x

[mcag236-B48] Tangney R , McInnes SJ, Dalziell EL, et al 2025. Defining the pyro-thermal niche: do seed traits, ecosystem type and phylogeny influence thermal thresholds in seeds with physical dormancy? The New Phytologist 246: 1567–1582. doi:10.1111/nph.70061 40084641

[mcag236-B49] Vázquez-Ramírez J , Venn SE. 2025. Snow, fire and drought: how alpine and treeline soil seed banks are affected by simulated climate change. Annals of Botany 135: 223–238. doi:10.1093/aob/mcad184 PMC11805938 38011645

[mcag236-B50] Verrall B , Pickering CM. 2019. Recovery of subalpine grasslands 15 years after landscape level fires. Australian Journal of Botany 67: 425–436. doi:10.1071/BT19020

[mcag236-B51] Virkkala A-M , Rogers BM, Watts JD, et al 2025. Wildfires offset the increasing but spatially heterogeneous Arctic–boreal CO_2_ uptake. Nature Climate Change 15: 188–195. doi:10.1038/s41558-024-02234-5

[mcag236-B52] Wang X , Ge W, Zhang M, et al 2024. Large and non-spherical seeds are less likely to form a persistent soil seed bank. Proceedings of the Royal Society of London: Series B, Biological Sciences 291: 20232764. doi:10.1098/rspb.2023.2764 PMC11285897 38864324

[mcag236-B53] Zaki E , Abedi M, Naqinezhad A. 2021. How fire history affects germination cues of three perennial grasses from the mountain steppes of Golestan National Park. Flora 280: 151835. doi:10.1016/j.flora.2021.151835

