# Supplementary material for: Fire as a regeneration filter in the Arctic: effects of heat and smoke on seed germination in 25 common plant species
Source: Ann Bot. 2026 Aug 14;138(5):1578–90. doi: 10.1093/aob/mcag236 (PMC13715022; doi:10.1093/aob/mcag236)
Supplement: Supplementary file 1 [file mcag236_supplementary_data.docx]

**Fire as a regeneration filter: contrasting effects of heat and smoke on Arctic seed germination**

Jerónimo Vázquez-Ramírez*, Margherita Tognela*, Natasha de Vere, Barbara Gawlak, Julia Kemppinen, Daniel Kepski, David Kniha, Simone Iris Lang, Petr Macek, Maija-Susanna Sujala, Otso Suominen, Anne Tolvanen, Brandon Samuel Whitley and Sergey Rosbakh.

*These authors contributed equally to this work.
Correspondence: Jerónimo Vázquez-Ramírez ([vasq@plen.ku.dk](mailto:vasq@plen.ku.dk)) | Margherita Tognela ([margherita.tognela01@universitadipavia.it](mailto:margherita.tognela01@universitadipavia.it)).

**The following supplementary materials are available:**

**Tables:**

- Table S1. Taxonomic identity, functional group, habitat, and collection metadata of the studied species.
- Table S2. Pairwise contrasts comparing the effects of low-heat, smoke, and combined low-heat + smoke treatments against the control on final germination percentage
- Table S3. Pairwise contrasts comparing the effects of low-heat, smoke, and combined low-heat + smoke treatments against the control on final germination speed
- Table S3. Pairwise contrasts comparing the effects of low-heat, smoke, and combined low-heat + smoke treatments against the control on final germination synchrony

**Figures:**

- Figure S1. Seed collection sites for all species included in the study.
- Figure S2. Final germination percentage (mean ± SE) for each species under control and high-temperature treatments.
- Figure S3. Species-specific effects of low heat, smoke, and their combination on final germination percentage.
- Figure S4. Species-specific effects of low heat, smoke, and their combination on germination speed.
- Figure S5. Species-specific effects of low heat, smoke, and their combination on germination synchrony.

**Code & Gemination data:**

- Raw germination counts and R code used to analyze them are available at <https://doi.org/10.5281/zenodo.18302947>

**Table S1.** Taxonomic identity, functional group, habitat, and collection metadata of the studied species.

| **Family** | **Species** | **Growth form** | **Functional group** | **Habitat** | **Collection site** | **Collection date** | **Collector** | **Coordinates** |
| --- | --- | --- | --- | --- | --- | --- | --- | --- |
| Asteraceae | *Taraxacum bracteatum* Dahlst. | Forb | Forb | Moist to mesic tundra and snowbed margins | Odindalen, Svalbard | 17.07.2023 | Petr Macek | 78.668242, 16.020826 |
| Betulaceae | *Betula nana* L. | Shrub or tree | Woody plant | Mesic to moist dwarf-shrub Arctic tundra | Kilpisjärvi Biological Station, Finland | 08.08.2023 | Maija Sujala, Oula Kalttopää | 69.044211, 20.80719 |
|  | *Betula pubescens* Ehrh. | Tree/ Tall shrub | Woody plant | Subarctic/alpine treeline birch forests | Kilpisjärvi Biological Station, Finland | 07.08.2023 | Maija Sujala | 69.040428, 20.814928 |
| Brassicaceae | *Arabis alpina* L. | Forb | Forb | Open, rocky alpine slopes and screes | Låktatjåkko Montain, Abisko, Sweden | 23.08.2023 | Sergey Rosbakh | 68.3987779, 18.4495201 |
|  | *Cochlearia groenlandica* L. | Forb | Forb | Saline Arctic coastal shores and beaches | Odindalen, Svalbard | 17.07.2023 | Petr Macek | 78.668242, 16.020826 |
| Caryophyllaceae | *Cerastium alpinum* L. | Forb | Forb | Dry, stony alpine ridges and fellfields | Polish Polar Station, Hornsund, Svalbard | 14.08.2023 | Daniel Kepski, Barbara Gawlak | 77.011528, 15.588269 |
|  | *Silene acaulis* (L.) Jacq. | Dwarf shrub | Woody plant | Dry, wind-exposed fellfields and ridges | Endalen, Svalbard | 23.08.2023 | Simone I. Lang | 78.112443, 15.454996 |
|  | *Silene involucrata* (Cham. & Schltdl.) Bocquet | Forb | Forb | Dry alpine screes and rocky slopes | Odindalen, Svalbard | 18.07.2023 | Petr Macek | 78.668242, 16.020826 |
| Cyperaceae | *Eriophorum scheuchzeri* Hoppe | Graminoid | Graminoid | Wet tundra, fens, and shallow wetlands | Adventdalen, Svalbard | 26.07.2023 | Simone I. Lang | 78.10308, 16.3427 |
| Ericaceae | *Cassiope tetragona* (L.) D.Don | Dwarf shrub | Woody plant | Dry, nutrient-poor high-Arctic heaths | Nuolja Mountain, Abisko, Sweden | 14.08.2024 | Sergey Rosbakh | 68.372377, 18.700501 |
|  | *Kalmia procumbens* (L.) Gift, Kron & P.F.Stevens ex Galasso, Banfi & F.Conti | Dwarf shrub | Woody plant | Dry, wind-exposed acidic fellfields | Kevo Research Station, Finland | 19.09.2023 | Elina Vainio | 69.82053, 27.12317 |
|  | *Phyllodoce caerulea* (L.) Bab. | Dwarf shrub | Woody plant | Acidic alpine heaths and ridges | Kilpisjärvi Biological Station, Finland | 23.08.2023 | Silja Veikkolainen and Maija Sujala | 69.044211, 20.80719 |
| Fabaceae | *Hedysarum alpinum* L. | Forb | Forb | Mesic tundra slopes and river terraces | Churchill Rocket Research Range, Canada | 11.08.2023 | Lara Grevstad | 58.751479, -93.859260 |
| Juncaceae | *Luzula confusa* Lindeb. | Graminoid | Graminoid | Moist to mesic tundra and snowbeds | Polish Polar Station, Hornsund, Svalbard | 18.08.2023 | Daniel Kepski, Barbara Gawlak | 77.00763, 15.52784 |
| Onagraceae | *Epilobium angustifolium* L. | Forb | Forb | Moist alpine tundra, snowbeds, and stream margins | Låktatjåkko, Abisko, Sweden | 18.08.2024 | Sergey Rosbakh | 68.381001, 18.439994 |
|  | *Epilobium anagallidifolium* Lam. | Forb | Forb | Disturbed, nutrient-rich post-fire and alluvial sites | Kallebrann, Sør-Varanger, Norway | 07.09.2023 | David Kniha | 69.456373, 30.033928 |
| Plantaginaceae | *Veronica alpina* L. | Forb | Forb | Moist alpine meadows and snowbeds | Latnjajaure, Sweden | 09.08.2023 | Wilhelm Osterman | 68.355836, 18.4945597 |
| Poaceae | *Anthoxanthum odoratum* L. | Graminoid | Graminoid | Dry, wind-exposed alpine heaths and ridges | Nuolja Mountain, Abisko, Sweden | 23.08.2023 | Sergey Rosbakh | 68.3696567, 18.7022515 |
|  | *Phleum alpinum* L. | Graminoid | Graminoid | Mesic, nutrient-rich alpine meadows and snowbeds | Låktatjåkko, Abisko, Sweden | 12.08.2024 | Sergey Rosbakh | 68.395045, 18.326951 |
| Polygonaceae | *Bistorta vivipara* (L.) Delarbre | Forb | Forb | Mesic tundra and snowbed communities | Qeqertarsuaq, Greenland | 10.08.2024 | Brandon Samuel Whitley | 69.253659, -53.528396 |
|  | *Oxyria digyna* (L.) Hill | Forb | Forb | Moist talus and recently deglaciated substrates | Odindalen, Svalbard | 17.07.2023 | Petr Macek | 78.668242, 16.020826 |
| Rosaceae | *Dryas integrifolia* Vahl | Dwarf shrub | Woody plant | Dry, calcareous Arctic heaths and ridges | Churchill Rocket Research Range, Canada | 31.07.2023 | Lara Grevstad | 58.734242, -93.810390 |
| Saxifragaceae | *Micranthes nivalis* (L.) Small | Forb | Forb | Moist rocky sites and meltwater channels | Polish Polar Station, Hornsund, Svalbard | 19.09.2023 | Daniel Kepski, Barbara Gawlak | 77.00763, 15.52784 |
|  | *Saxifraga cernua* L. | Forb | Forb | Moist, late-snow Arctic habitats | Polish Polar Station, Hornsund, Svalbard | 28.08.2023 | Daniel Kepski, Barbara Gawlak | 77.00763, 15.52784 |
|  | *Saxifraga cespitosa* L. | Forb | Forb | Dry, rocky alpine ridges and screes | Odindalen, Svalbard | 17.07.2023 | Petr Macek | 78.668242, 16.020826 |

**Table S2**. Pairwise contrasts comparing the effects of low-heat, smoke, and combined low-heat + smoke treatments against the control on **final germination percentage** for 25 Arctic plant species. Values shown are odds ratios with 95% confidence intervals and associated *p*-values.

| **Species name** | **contrast** | **odds.ratio** | **SE** | **asymp.LCL** | **asymp.UCL** | **p.value** |
| --- | --- | --- | --- | --- | --- | --- |
| *Anthoxanthum odoratum* | Low-heat / Control | 0.820 | 0.342 | 0.306 | 2.195 | 0.905 |
|  | Smoke / Control | 1.311 | 0.535 | 0.501 | 3.434 | 0.816 |
|  | Low-heat + Smoke / Control | 1.237 | 0.502 | 0.474 | 3.225 | 0.885 |
| *Arabis alpina* | Low-heat / Control | 0.966 | 0.357 | 0.403 | 2.311 | 0.997 |
|  | Smoke / Control | 1.435 | 0.538 | 0.593 | 3.474 | 0.636 |
|  | Low-heat + Smoke / Control | 0.765 | 0.280 | 0.322 | 1.817 | 0.779 |
| *Betula nana* | Low-heat / Control | 0.612 | 0.367 | 0.148 | 2.524 | 0.727 |
|  | Smoke / Control | 0.650 | 0.392 | 0.156 | 2.701 | 0.789 |
|  | Low-heat + Smoke / Control | 0.578 | 0.345 | 0.141 | 2.368 | 0.665 |
| *Betula pubescens* | Low-heat / Control | 0.897 | 0.359 | 0.349 | 2.304 | 0.970 |
|  | Smoke / Control | 1.448 | 0.579 | 0.564 | 3.720 | 0.660 |
|  | Low-heat + Smoke / Control | 0.850 | 0.343 | 0.328 | 2.202 | 0.933 |
| *Bistorta vivipara* | Low-heat / Control | 2.074 | 2.601 | 0.108 | 39.983 | 0.858 |
|  | Smoke / Control | 2.148 | 2.693 | 0.112 | 41.363 | 0.844 |
|  | Low-heat + Smoke / Control | 2.148 | 2.693 | 0.112 | 41.363 | 0.844 |
| *Cassiope tetragona* | Low-heat / Control | 1.899 | 0.894 | 0.626 | 5.765 | 0.385 |
|  | Smoke / Control | 4.186 | 2.811 | 0.858 | 20.416 | 0.088 |
|  | Low-heat + Smoke / Control | 0.501 | 0.203 | 0.193 | 1.300 | 0.214 |
| *Cerastium alpinum* | Low-heat / Control | 0.878 | 0.340 | 0.353 | 2.188 | 0.954 |
|  | Smoke / Control | 0.785 | 0.297 | 0.321 | 1.915 | 0.828 |
|  | Low-heat + Smoke / Control | 1.322 | 0.518 | 0.525 | 3.333 | 0.789 |
| *Cochlearia groenlandica* | Low-heat / Control | 0.538 | 0.242 | 0.186 | 1.557 | 0.378 |
|  | Smoke / Control | 0.686 | 0.309 | 0.237 | 1.982 | 0.714 |
|  | Low-heat + Smoke / Control | 0.891 | 0.426 | 0.289 | 2.749 | 0.977 |
| *Dryas integrifolia* | Low-heat / Control | 0.627 | 0.286 | 0.214 | 1.842 | 0.598 |
|  | Smoke / Control | 0.837 | 0.401 | 0.271 | 2.592 | 0.943 |
|  | Low-heat + Smoke / Control | 3.335 | 1.934 | 0.849 | 13.098 | 0.099 |
| *Epilobium anagallidifolium* | Low-heat / Control | 0.481 | 0.438 | 0.056 | 4.117 | 0.735 |
|  | Smoke / Control | 0.369 | 0.326 | 0.046 | 2.967 | 0.529 |
|  | Low-heat + Smoke / Control | 0.560 | 0.469 | 0.078 | 4.040 | 0.801 |
| *Epilobium angustifolium* | Low-heat / Control | 4119830191 | NA | NA | NA | NA |
|  | Smoke / Control | 1.528 | NA | NA | NA | NA |
|  | Low-heat + Smoke / Control | 3.278 | NA | NA | NA | NA |
| *Eriophorum scheuchzeri* | Low-heat / Control | 0.675 | 0.416 | 0.158 | 2.893 | 0.831 |
|  | Smoke / Control | 5.273 | 5.861 | 0.383 | 72.604 | 0.313 |
|  | Low-heat + Smoke / Control | 2.409 | 2.068 | 0.318 | 18.263 | 0.596 |
| *Hedysarum alpinum* | Low-heat / Control | 1.937 | 0.872 | 0.670 | 5.601 | 0.326 |
|  | Smoke / Control | 1.823 | 0.828 | 0.625 | 5.322 | 0.408 |
|  | Low-heat + Smoke / Control | 3.178 | 1.382 | 1.139 | 8.867 | 0.022 |
| *Kalmia procumbens* | Low-heat / Control | 0.769 | 0.293 | 0.313 | 1.890 | 0.803 |
|  | Smoke / Control | 1.661 | 0.616 | 0.693 | 3.982 | 0.382 |
|  | Low-heat + Smoke / Control | 1.759 | 0.642 | 0.743 | 4.162 | 0.287 |
| *Luzula confusa* | Low-heat / Control | 1.565 | 0.581 | 0.652 | 3.759 | 0.479 |
|  | Smoke / Control | 1.222 | 0.447 | 0.515 | 2.899 | 0.874 |
|  | Low-heat + Smoke / Control | 1.269 | 0.467 | 0.533 | 3.024 | 0.825 |
| *Micranthes nivalis* | Low-heat / Control | 0.813 | 0.642 | 0.126 | 5.242 | 0.972 |
|  | Smoke / Control | 1.141 | 0.959 | 0.157 | 8.287 | 0.990 |
|  | Low-heat + Smoke / Control | 1.750 | 1.635 | 0.193 | 15.858 | 0.850 |
| *Oxyria digyna* | Low-heat / Control | 0.646 | 0.380 | 0.162 | 2.586 | 0.772 |
|  | Smoke / Control | 1.488 | 1.091 | 0.264 | 8.394 | 0.877 |
|  | Low-heat + Smoke / Control | 0.542 | 0.310 | 0.141 | 2.093 | 0.567 |
| *Phleum alpinum* | Low-heat / Control | 0.523 | 0.369 | 0.099 | 2.768 | 0.665 |
|  | Smoke / Control | 3.538 | 4.020 | 0.242 | 51.650 | 0.539 |
|  | Low-heat + Smoke / Control | 2.615 | 2.980 | 0.178 | 38.481 | 0.711 |
| *Phyllodoce caerulea* | Low-heat / Control | 0.727 | 0.508 | 0.140 | 3.781 | 0.913 |
|  | Smoke / Control | 0.630 | 0.425 | 0.129 | 3.091 | 0.805 |
|  | Low-heat + Smoke / Control | 0.317 | 0.196 | 0.074 | 1.359 | 0.159 |
| *Saxifraga cernua* | Low-heat / Control | 0.735 | 0.446 | 0.175 | 3.076 | 0.892 |
|  | Smoke / Control | 0.972 | 0.594 | 0.230 | 4.111 | 0.999 |
|  | Low-heat + Smoke / Control | 0.821 | 0.494 | 0.198 | 3.396 | 0.956 |
| *Saxifraga cespitosa* | Low-heat / Control | 11.732 | 2060473.720 | 2.2204469031e-16 | Inf | 1.000 |
|  | Smoke / Control | 6.377445721386e-11 | 3.162613987695e-06 | 2.2206049031e-16 | Inf | 1.000 |
|  | Low-heat + Smoke / Control | 11.367 | 1955973.262 | 2.2204469031e-16 | Inf | 1.000 |
| *Silene acaulis* | Low-heat / Control | 1.072 | 0.401 | 0.444 | 2.590 | 0.986 |
|  | Smoke / Control | 1.432 | 0.553 | 0.576 | 3.561 | 0.657 |
|  | Low-heat + Smoke / Control | 1.634 | 0.633 | 0.655 | 4.074 | 0.441 |
| *Silene furcata* | Low-heat / Control | 0.661 | 0.524 | 0.102 | 4.297 | 0.886 |
|  | Smoke / Control | 0.450 | 0.332 | 0.079 | 2.568 | 0.559 |
|  | Low-heat + Smoke / Control | 0.444 | 0.328 | 0.078 | 2.538 | 0.548 |
| *Taraxacum bracteatum* | Low-heat / Control | 0.429 | 0.381 | 0.053 | 3.490 | 0.642 |
|  | Smoke / Control | 0.643 | 0.600 | 0.071 | 5.806 | 0.906 |
|  | Low-heat + Smoke / Control | 0.456 | 0.404 | 0.056 | 3.698 | 0.685 |
| *Veronica alpina* | Low-heat / Control | 0.923 | 0.493 | 0.262 | 3.255 | 0.991 |
|  | Smoke / Control | 0.865 | 0.466 | 0.243 | 3.079 | 0.971 |
|  | Low-heat + Smoke / Control | 1.275 | 0.704 | 0.347 | 4.689 | 0.919 |

Note: For some species, extremely large or small odds ratios, large standard errors, or infinite confidence intervals resulted from complete or near-complete separation in the binomial models due to consistently high or low germination across treatments. These estimates should therefore be interpreted with caution and considered alongside with the observed germination percentages presented in Figure S3.

**Table S3.** Pairwise contrasts comparing the effects of low-heat, smoke, and combined low-heat + smoke treatments against the control on **germination speed** for 25 Arctic plant species. Values shown are estimates with 95% confidence intervals and associated *p*-values (significant values color coded: green = increase and red = decrease in germination speed)

| **Species_full** | **contrast** | **estimate** | **SE** | **asymp.LCL** | **asymp.UCL** | **p.value** |
| --- | --- | --- | --- | --- | --- | --- |
| *Anthoxanthum odoratum* | Low-heat / Control | -0.081 | 0.135 | -0.504 | 0.342 | 0.858 |
|  | Smoke / Control | 0.097 | 0.135 | -0.326 | 0.520 | 0.797 |
|  | Low-heat + Smoke / Control | -0.031 | 0.135 | -0.454 | 0.392 | 0.979 |
| *Arabis alpina* | Low-heat / Control | -0.119 | 0.036 | -0.233 | -0.006 | 0.042 |
|  | Smoke / Control | 0.046 | 0.036 | -0.068 | 0.160 | 0.496 |
|  | Low-heat + Smoke / Control | 0.062 | 0.036 | -0.052 | 0.176 | 0.306 |
| *Betula nana* | Low-heat / Control | 0.157 | 0.081 | -0.097 | 0.410 | 0.229 |
|  | Smoke / Control | 0.243 | 0.081 | -0.011 | 0.497 | 0.059 |
|  | Low-heat + Smoke / Control | 0.424 | 0.081 | 0.171 | 0.678 | 0.005 |
| *Betula pubescens* | Low-heat / Control | -0.002 | 0.099 | -0.314 | 0.310 | 1.000 |
|  | Smoke / Control | -0.016 | 0.099 | -0.329 | 0.296 | 0.990 |
|  | Low-heat + Smoke / Control | 0.089 | 0.099 | -0.224 | 0.401 | 0.707 |
| *Bistorta vivipara* | Low-heat / Control | -0.033 | 0.090 | -0.317 | 0.251 | 0.947 |
|  | Smoke / Control | 0.193 | 0.090 | -0.091 | 0.477 | 0.179 |
|  | Low-heat + Smoke / Control | 0.145 | 0.090 | -0.139 | 0.429 | 0.346 |
| *Cassiope tetragona* | Low-heat / Control | -0.106 | 0.044 | -0.254 | 0.041 | 0.142 |
|  | Smoke / Control | -0.013 | 0.053 | -0.182 | 0.156 | 0.976 |
|  | Low-heat + Smoke / Control | -0.038 | 0.044 | -0.185 | 0.110 | 0.732 |
| *Cerastium alpinum* | Low-heat / Control | 0.042 | 0.098 | -0.268 | 0.351 | 0.928 |
|  | Smoke / Control | 0.038 | 0.098 | -0.271 | 0.348 | 0.939 |
|  | Low-heat + Smoke / Control | 0.141 | 0.098 | -0.168 | 0.451 | 0.418 |
| *Cochlearia groenlandica* | Low-heat / Control | 0.055 | 0.132 | -0.359 | 0.469 | 0.931 |
|  | Smoke / Control | 0.194 | 0.132 | -0.220 | 0.608 | 0.402 |
|  | Low-heat + Smoke / Control | 0.099 | 0.132 | -0.315 | 0.513 | 0.784 |
| *Dryas integrifolia* | Low-heat / Control | -0.290 | 0.084 | -0.555 | -0.025 | 0.035 |
|  | Smoke / Control | 0.311 | 0.084 | 0.045 | 0.576 | 0.026 |
|  | Low-heat + Smoke / Control | 0.199 | 0.084 | -0.067 | 0.464 | 0.136 |
| *Epilobium anagallidifolium* | Low-heat / Control | -0.181 | 0.101 | -0.497 | 0.136 | 0.275 |
|  | Smoke / Control | 0.098 | 0.101 | -0.218 | 0.415 | 0.660 |
|  | Low-heat + Smoke / Control | 0.051 | 0.101 | -0.265 | 0.368 | 0.896 |
| *Epilobium angustifolium* | Low-heat / Control | -0.097 | 0.124 | -0.487 | 0.292 | 0.766 |
|  | Smoke / Control | -0.192 | 0.124 | -0.582 | 0.197 | 0.367 |
|  | Low-heat + Smoke / Control | 0.034 | 0.124 | -0.356 | 0.424 | 0.970 |
| *Eriophorum scheuchzeri* | Low-heat / Control | -0.054 | 0.035 | -0.163 | 0.055 | 0.361 |
|  | Smoke / Control | 0.377 | 0.035 | 0.268 | 0.485 | <0.001 |
|  | Low-heat + Smoke / Control | 0.187 | 0.035 | 0.079 | 0.296 | 0.004 |
| *Hedysarum alpinum* | Low-heat / Control | 0.023 | 0.276 | -0.844 | 0.890 | 0.997 |
|  | Smoke / Control | -0.263 | 0.276 | -1.130 | 0.603 | 0.672 |
|  | Low-heat + Smoke / Control | 0.219 | 0.276 | -0.648 | 1.086 | 0.761 |
| *Kalmia procumbens* | Low-heat / Control | -0.032 | 0.026 | -0.114 | 0.049 | 0.511 |
|  | Smoke / Control | 0.016 | 0.026 | -0.065 | 0.097 | 0.851 |
|  | Low-heat + Smoke / Control | 0.064 | 0.026 | -0.017 | 0.145 | 0.117 |
| *Luzula confusa* | Low-heat / Control | 0.285 | 0.064 | 0.083 | 0.487 | 0.011 |
|  | Smoke / Control | 0.157 | 0.064 | -0.045 | 0.359 | 0.121 |
|  | Low-heat + Smoke / Control | 0.153 | 0.064 | -0.049 | 0.355 | 0.130 |
| *Micranthes nivalis* | Low-heat / Control | -0.280 | 0.025 | -0.360 | -0.201 | <0.001 |
|  | Smoke / Control | -0.101 | 0.025 | -0.180 | -0.021 | 0.018 |
|  | Low-heat + Smoke / Control | -0.047 | 0.025 | -0.127 | 0.032 | 0.248 |
| *Oxyria digyna* | Low-heat / Control | 0.221 | 0.123 | -0.165 | 0.607 | 0.273 |
|  | Smoke / Control | 0.171 | 0.123 | -0.215 | 0.556 | 0.440 |
|  | Low-heat + Smoke / Control | 0.082 | 0.123 | -0.304 | 0.467 | 0.826 |
| *Phleum alpinum* | Low-heat / Control | -0.142 | 0.040 | -0.269 | -0.015 | 0.032 |
|  | Smoke / Control | -0.028 | 0.040 | -0.155 | 0.099 | 0.808 |
|  | Low-heat + Smoke / Control | -0.198 | 0.040 | -0.324 | -0.071 | 0.007 |
| *Phyllodoce caerulea* | Low-heat / Control | -0.064 | 0.030 | -0.160 | 0.031 | 0.185 |
|  | Smoke / Control | -0.009 | 0.030 | -0.105 | 0.086 | 0.961 |
|  | Low-heat + Smoke / Control | 0.000 | 0.030 | -0.095 | 0.096 | 1.000 |
| *Saxifraga cernua* | Low-heat / Control | -0.084 | 0.217 | -0.768 | 0.600 | 0.940 |
|  | Smoke / Control | 0.228 | 0.217 | -0.455 | 0.912 | 0.618 |
|  | Low-heat + Smoke / Control | -0.056 | 0.217 | -0.740 | 0.628 | 0.974 |
| *Saxifraga cespitosa* | Low-heat / Control | -0.155 | 0.027 | -0.240 | -0.071 | 0.003 |
|  | Smoke / Control | -0.099 | 0.027 | -0.184 | -0.015 | 0.026 |
|  | Low-heat + Smoke / Control | -0.056 | 0.027 | -0.140 | 0.029 | 0.196 |
| *Silene acaulis* | Low-heat / Control | -0.108 | 0.158 | -0.606 | 0.389 | 0.816 |
|  | Smoke / Control | 0.274 | 0.158 | -0.223 | 0.771 | 0.296 |
|  | Low-heat + Smoke / Control | 0.238 | 0.158 | -0.259 | 0.735 | 0.386 |
| *Silene furcata* | Low-heat / Control | 0.155 | 0.039 | 0.032 | 0.277 | 0.019 |
|  | Smoke / Control | 0.175 | 0.039 | 0.053 | 0.298 | 0.011 |
|  | Low-heat + Smoke / Control | 0.182 | 0.039 | 0.060 | 0.305 | 0.009 |
| *Taraxacum bracteatum* | Low-heat / Control | -0.172 | 0.118 | -0.543 | 0.198 | 0.406 |
|  | Smoke / Control | 0.249 | 0.118 | -0.121 | 0.620 | 0.184 |
|  | Low-heat + Smoke / Control | 0.699 | 0.118 | 0.329 | 1.070 | 0.003 |
| *Veronica alpina* | Low-heat / Control | -0.196 | 0.103 | -0.518 | 0.126 | 0.238 |
|  | Smoke / Control | 0.088 | 0.103 | -0.234 | 0.410 | 0.725 |
|  | Low-heat + Smoke / Control | -0.094 | 0.103 | -0.416 | 0.229 | 0.695 |

**Table S4.** Pairwise contrasts comparing the effects of low-heat, smoke, and combined low-heat + smoke treatments against the control on **germination synchrony** for 25 Arctic plant species. Values shown are estimates with 95% confidence intervals and associated *p*-values (significant values color coded: green = increase and red = decrease in synchrony)

| **Species_full** | **contrast** | **estimate** | **SE** | **asymp.LCL** | **asymp.UCL** | **p.value** |
| --- | --- | --- | --- | --- | --- | --- |
| *Anthoxanthum odoratum* | Low-heat / Control | -0.021 | 0.108 | -0.361 | 0.319 | 0.858 |
|  | Smoke / Control | -0.124 | 0.108 | -0.464 | 0.216 | 0.797 |
|  | Low-heat + Smoke / Control | -0.124 | 0.108 | -0.464 | 0.216 | 0.979 |
| *Arabis alpina* | Low-heat / Control | -0.270 | 0.086 | -0.540 | 0.000 | 0.042 |
|  | Smoke / Control | 0.137 | 0.086 | -0.133 | 0.406 | 0.496 |
|  | Low-heat + Smoke / Control | 0.184 | 0.086 | -0.085 | 0.454 | 0.306 |
| *Betula nana* | Low-heat / Control | 0.078 | 0.316 | -0.915 | 1.070 | 0.229 |
|  | Smoke / Control | -0.144 | 0.316 | -1.137 | 0.848 | 0.059 |
|  | Low-heat + Smoke / Control | -0.089 | 0.316 | -1.081 | 0.904 | 0.005 |
| *Betula pubescens* | Low-heat / Control | 0.143 | 0.137 | -0.286 | 0.572 | 1.000 |
|  | Smoke / Control | -0.012 | 0.137 | -0.442 | 0.417 | 0.990 |
|  | Low-heat + Smoke / Control | 0.180 | 0.137 | -0.249 | 0.610 | 0.707 |
| *Bistorta vivipara* | Low-heat / Control | 0.396 | 0.093 | 0.103 | 0.690 | 0.947 |
|  | Smoke / Control | 0.131 | 0.093 | -0.162 | 0.425 | 0.179 |
|  | Low-heat + Smoke / Control | 0.124 | 0.093 | -0.169 | 0.417 | 0.346 |
| *Cassiope tetragona* | Low-heat / Control | 0.017 | 0.062 | -0.190 | 0.225 | 0.142 |
|  | Smoke / Control | 0.136 | 0.074 | -0.101 | 0.374 | 0.976 |
|  | Low-heat + Smoke / Control | 0.092 | 0.062 | -0.116 | 0.299 | 0.732 |
| *Cerastium alpinum* | Low-heat / Control | -0.125 | 0.067 | -0.337 | 0.086 | 0.928 |
|  | Smoke / Control | -0.104 | 0.067 | -0.316 | 0.108 | 0.939 |
|  | Low-heat + Smoke / Control | -0.071 | 0.067 | -0.283 | 0.140 | 0.418 |
| *Cochlearia groenlandica* | Low-heat / Control | 0.002 | 0.167 | -0.522 | 0.526 | 0.931 |
|  | Smoke / Control | 0.224 | 0.167 | -0.300 | 0.748 | 0.402 |
|  | Low-heat + Smoke / Control | 0.108 | 0.167 | -0.416 | 0.632 | 0.784 |
| *Dryas integrifolia* | Low-heat / Control | -0.002 | 0.057 | -0.181 | 0.177 | 0.035 |
|  | Smoke / Control | 0.003 | 0.057 | -0.176 | 0.182 | 0.026 |
|  | Low-heat + Smoke / Control | 0.001 | 0.057 | -0.178 | 0.180 | 0.136 |
| *Epilobium anagallidifolium* | Low-heat / Control | 0.089 | 0.091 | -0.196 | 0.375 | 0.275 |
|  | Smoke / Control | -0.003 | 0.091 | -0.289 | 0.283 | 0.660 |
|  | Low-heat + Smoke / Control | -0.125 | 0.091 | -0.411 | 0.161 | 0.896 |
| *Epilobium angustifolium* | Low-heat / Control | -0.045 | 0.035 | -0.154 | 0.063 | 0.766 |
|  | Smoke / Control | -0.104 | 0.035 | -0.212 | 0.005 | 0.367 |
|  | Low-heat + Smoke / Control | 0.163 | 0.035 | 0.054 | 0.271 | 0.970 |
| *Eriophorum scheuchzeri* | Low-heat / Control | -0.014 | 0.069 | -0.232 | 0.204 | 0.361 |
|  | Smoke / Control | 0.315 | 0.069 | 0.097 | 0.533 | <0.001 |
|  | Low-heat + Smoke / Control | -0.026 | 0.069 | -0.244 | 0.192 | 0.004 |
| *Hedysarum alpinum* | Low-heat / Control | 0.200 | 0.071 | -0.024 | 0.424 | 0.997 |
|  | Smoke / Control | 0.033 | 0.071 | -0.191 | 0.258 | 0.672 |
|  | Low-heat + Smoke / Control | 0.141 | 0.071 | -0.083 | 0.365 | 0.761 |
| *Kalmia procumbens* | Low-heat / Control | 0.065 | 0.115 | -0.296 | 0.426 | 0.511 |
|  | Smoke / Control | -0.113 | 0.115 | -0.474 | 0.248 | 0.851 |
|  | Low-heat + Smoke / Control | -0.185 | 0.115 | -0.546 | 0.176 | 0.117 |
| *Luzula confusa* | Low-heat / Control | 0.324 | 0.086 | 0.054 | 0.595 | 0.011 |
|  | Smoke / Control | 0.155 | 0.086 | -0.115 | 0.426 | 0.121 |
|  | Low-heat + Smoke / Control | 0.078 | 0.086 | -0.193 | 0.348 | 0.130 |
| *Micranthes nivalis* | Low-heat / Control | -0.182 | 0.100 | -0.498 | 0.133 | <0.001 |
|  | Smoke / Control | -0.286 | 0.100 | -0.601 | 0.030 | 0.018 |
|  | Low-heat + Smoke / Control | -0.103 | 0.100 | -0.419 | 0.212 | 0.248 |
| *Oxyria digyna* | Low-heat / Control | 0.238 | 0.085 | -0.029 | 0.506 | 0.273 |
|  | Smoke / Control | 0.081 | 0.085 | -0.187 | 0.348 | 0.440 |
|  | Low-heat + Smoke / Control | -0.137 | 0.085 | -0.404 | 0.131 | 0.826 |
| *Phleum alpinum* | Low-heat / Control | -0.370 | 0.102 | -0.689 | -0.051 | 0.032 |
|  | Smoke / Control | -0.130 | 0.102 | -0.449 | 0.190 | 0.808 |
|  | Low-heat + Smoke / Control | -0.387 | 0.102 | -0.706 | -0.067 | 0.007 |
| *Phyllodoce caerulea* | Low-heat / Control | 0.058 | 0.049 | -0.098 | 0.213 | 0.185 |
|  | Smoke / Control | 0.018 | 0.049 | -0.137 | 0.173 | 0.961 |
|  | Low-heat + Smoke / Control | 0.020 | 0.049 | -0.135 | 0.175 | 1.000 |
| *Saxifraga cernua* | Low-heat / Control | -0.052 | 0.144 | -0.504 | 0.399 | 0.940 |
|  | Smoke / Control | -0.010 | 0.144 | -0.462 | 0.441 | 0.618 |
|  | Low-heat + Smoke / Control | -0.160 | 0.144 | -0.611 | 0.291 | 0.974 |
| *Saxifraga cespitosa* | Low-heat / Control | 0.298 | 0.061 | 0.105 | 0.492 | 0.003 |
|  | Smoke / Control | 0.114 | 0.061 | -0.079 | 0.307 | 0.026 |
|  | Low-heat + Smoke / Control | -0.064 | 0.061 | -0.257 | 0.130 | 0.196 |
| *Silene acaulis* | Low-heat / Control | 0.068 | 0.043 | -0.066 | 0.202 | 0.816 |
|  | Smoke / Control | 0.084 | 0.043 | -0.050 | 0.218 | 0.296 |
|  | Low-heat + Smoke / Control | 0.102 | 0.043 | -0.032 | 0.236 | 0.386 |
| *Silene furcata* | Low-heat / Control | 0.154 | 0.027 | 0.070 | 0.237 | 0.019 |
|  | Smoke / Control | 0.270 | 0.027 | 0.187 | 0.354 | 0.011 |
|  | Low-heat + Smoke / Control | 0.234 | 0.027 | 0.151 | 0.318 | 0.009 |
| *Taraxacum bracteatum* | Low-heat / Control | -0.053 | 0.102 | -0.373 | 0.266 | 0.406 |
|  | Smoke / Control | 0.163 | 0.102 | -0.156 | 0.482 | 0.184 |
|  | Low-heat + Smoke / Control | 0.516 | 0.102 | 0.196 | 0.835 | 0.003 |
| *Veronica alpina* | Low-heat / Control | -0.300 | 0.188 | -0.891 | 0.291 | 0.238 |
|  | Smoke / Control | -0.100 | 0.188 | -0.691 | 0.491 | 0.725 |
|  | Low-heat + Smoke / Control | -0.294 | 0.188 | -0.884 | 0.297 | 0.695 |

**Figure S1.** Geographic distribution of the 25 Arctic seed collection sites. Colours represent the functional groups of the sampled species (forbs, graminoids and woody plants).

**Figure S2**. Final germination percentage (mean ± SE) for each species under control and high-temperature treatments. Most species exhibited complete or near-complete germination failure under high heat, indicating extreme sensitivity to short-duration, high-intensity thermal exposure. Only three species (*A. odoratum*, *E. angustifolium*, and *P. alpinum*) showed any appreciable germination under high temperature, revealing pronounced interspecific variation in heat tolerance. *Silene acaulis* = *Silene involucrata* (Cham. & Schltdl.) Bocquet

**Figure S3.** Species-specific responses of final germination percentage (FGP) to low-heat, smoke, and combined smoke × low-heat treatments across 25 Arctic plant species*.* Bars show mean FGP ± SE per treatment and species. Across species, treatment effects were generally weak or absent, with only *Hedysarum alpinum* exhibiting a significant increase in germination under the Smoke × Low-heat treatment relative to Control. No other species showed statistically detectable treatment–control difference/ *Silene acaulis* = *Silene involucrata* (Cham. & Schltdl.) Bocquet

**Figure S4**. Species-specific responses of seed germination speed to low-heat, smoke, and their combination. Mean germination speed (± SE, back-transformed from log-scale) is shown for each treatment within 25 Arctic plant species. While many species exhibit minimal shifts in germination speed across treatments, a subset of taxa shows pronounced changes: in some species low-heat reduces germination speed, whereas in others smoke and Smoke × Low-heat accelerate germination relative to the control. Significant differences between treatments and the control (p < 0.05, Dunnett-adjusted tests from species-specific mixed models) occur in 10 of the 25 species, highlighting that smoke and mild heating can either delay or advance germination in a strongly species-dependent manner (Table S3). *Silene acaulis* = *Silene involucrata* (Cham. & Schltdl.) Bocquet

**Figure S5**. Species-specific effects of low heat, smoke, and their combination on germination synchrony. Mean germination synchrony (± SE) per treatment is shown for each of the 25 Arctic plant species, based on dish-level estimates. While most species exhibit broadly similar synchrony across treatments, mixed-effects models with Dunnett-adjusted contrasts against the control identify a subset in which treatments affected the temporal concentration of germination (see Table S4). *Silene acaulis* = *Silene involucrata* (Cham. & Schltdl.) Bocquet
